# Small but Crucial: The Novel Small Heat Shock Protein Hsp21 Mediates Stress Adaptation and Virulence in *Candida albicans*

**DOI:** 10.1371/journal.pone.0038584

**Published:** 2012-06-07

**Authors:** François L. Mayer, Duncan Wilson, Ilse D. Jacobsen, Pedro Miramón, Silvia Slesiona, Iryna M. Bohovych, Alistair J. P. Brown, Bernhard Hube

**Affiliations:** 1 Department of Microbial Pathogenicity Mechanisms, Hans-Knoell-Institute, Jena, Germany; 2 Department of Microbial Biochemistry and Physiology, Hans-Knoell-Institute, Jena, Germany; 3 Aberdeen Fungal Group, Institute of Medical Sciences, University of Aberdeen, Foresterhill, Aberdeen, United Kingdom; 4 Center for Sepsis Control and Care, Universitätsklinikum Jena, Jena, Germany; 5 Friedrich Schiller University, Jena, Germany; New Jersey Medical School, University of Medicine and Dentistry of New Jersey, United States of America

## Abstract

Small heat shock proteins (sHsps) have multiple cellular functions. However, the biological function of sHsps in pathogenic microorganisms is largely unknown. In the present study we identified and characterized the novel sHsp Hsp21 of the human fungal pathogen *Candida albicans*. Using a reverse genetics approach we demonstrate the importance of Hsp21 for resistance of *C. albicans* to specific stresses, including thermal and oxidative stress. Furthermore, a *hsp21*Δ/Δ mutant was defective in invasive growth and formed significantly shorter filaments compared to the wild type under various filament-inducing conditions. Although adhesion to and invasion into human-derived endothelial and oral epithelial cells was unaltered, the *hsp21*Δ/Δ mutant exhibited a strongly reduced capacity to damage both cell lines. Furthermore, Hsp21 was required for resisting killing by human neutrophils. Measurements of intracellular levels of stress protective molecules demonstrated that Hsp21 is involved in both glycerol and glycogen regulation and plays a major role in trehalose homeostasis in response to elevated temperatures. Mutants defective in trehalose and, to a lesser extent, glycerol synthesis phenocopied *HSP21* deletion in terms of increased susceptibility to environmental stress, strongly impaired capacity to damage epithelial cells and increased sensitivity to the killing activities of human primary neutrophils. Via systematic analysis of the three main *C. albicans* stress-responsive kinases (Mkc1, Cek1, Hog1) under a range of stressors, we demonstrate Hsp21-dependent phosphorylation of Cek1 in response to elevated temperatures. Finally, the *hsp21*Δ/Δ mutant displayed strongly attenuated virulence in two *in vivo* infection models. Taken together, Hsp21 mediates adaptation to specific stresses via fine-tuning homeostasis of compatible solutes and activation of the Cek1 pathway, and is crucial for multiple stages of *C. albicans* pathogenicity. Hsp21 therefore represents the first reported example of a small heat shock protein functioning as a virulence factor in a eukaryotic pathogen.

## Introduction

The heat shock response is an ancient and conserved reaction of living organisms to stressful conditions such as an elevation in temperature, oxidative stress or starvation [1]. Such stresses can result in protein unfolding and nonspecific aggregation, ultimately leading to cell death. In order to counteract this detrimental fate, cells synthesise so-called heat shock proteins (Hsps) [2]. These specialized proteins act as chaperones and prevent unfolding and aggregation of proteins by binding to their clients and stabilizing them [3]. There are five major families of Hsps [3], [4]; four of them - Hsp100s, Hsp90s, Hsp70s and Hsp60s - consist of ATP-dependent high-molecular-mass Hsps, while the fifth family - the small heat shock proteins (sHsps) - consist of ATP-independent low-molecular-mass Hsps with sizes ranging from 12 to 42 kDa [5]. The higher molecular mass Hsps are highly conserved amongst species and most of them are important for protein quality control procedures under both non-stress and stress conditions.

In contrast, sHsps display less sequence conservation between species and have been shown to be mainly expressed under stress conditions [6]. However, all sHsps share a central α-crystallin domain, which is named after the human lenticular protein α-crystallin. In the human eye, α-crystallin prevents protein aggregation and concomitant cataract formation [7], [8]. The sHsp α-crystallin domain is flanked by variable N- and C-terminal domains [6], [9]. On the transcriptional level, regulation of Hsps occurs through heat shock elements (HSEs), defined repeats of distinct nucleotide triplets [10], [11], [12].

In the last decades the large Hsps have been subject to more intensive study than the sHsps. Importantly, several investigations have demonstrated a connection between Hsps of pathogenic microorganisms and their virulence potential [13], [14], [15], [16], [17], [18], [19], [20], including Hsp90 [21] and Hsp70 [22] in the human fungal pathogen *Candida albicans*. In the non-pathogenic yeast *Saccharomyces cerevisiae* the sHSP HSp26 has unexpectedly been demonstrated not to be required for growth at elevated temperatures, nor for thermotolerance, spore devolpment, or germination [23], despite the fact that it accumulates in the cells during thermal and other forms of stress as a result of transcriptional derepression [24]. The sHsp Hsp12 is strongly upregulated (several 100-folds) in response to stress [25]. In contrast to ScHsp26 however, Hsp12 is required for growth/survival of a variety of stress conditions, and maintenance of normal cell morphology [25].

To the best of our knowledge, the role of sHsps in microbial pathogenicity has only been described for two bacteria so far, the Gram-positive human pathogenic bacterium *Mycobacterium tuberculosis* [26] and the Gram-negative plant pathogenic bacterium *Agrobacterium tumefaciens* [27], [28].

As yet only three sHSPs - Hsp10, Hsp12 and Hsp30/Hsp31 - have been identified in *C. albicans* (Table 1). Of these only Hsp12 has been characterized on a transcriptional level. RNA hybridization analyses demonstrated the co-regulation of *HSP12* by environmental pH and CO_2_ in this fungus [29]. The function of Hsp10 and Hsp30/Hsp31 remains unknown. On the other hand, their counterparts in *Saccharomyces cerevisiae* as well as the additional sHSPs ScHsp26, ScHsp40 and ScHsp42, have been investigated [25], [30], [31], [32], [33], [34], [35]. One of the key differences between these two species is that *C. albicans* is a major opportunistic fungal pathogen of humans.

**Table 1:** Small heat shock proteins in *Candida albicans* and *Saccharomyces cerevisiae*.

*C. albicans*	*S. cerevisiae*	*Function in C. albicans*	*Function in S. cerevisiae*	*Homology (% identity)*
Hsp10	Hsp10	unknown	inhibits ATPase activity of Hsp60 [30]	57
Hsp12	Hsp12	unknown	protects membranes from desiccation [25], [31]	44
orf19.822 (21 kDa sHsp)	–	mediates stress adaptation and virulence (This work)	–	–
–	Hsp26	–	suppresses unfolded protein aggregation [32]	–
Hsp30/Hsp31	Hsp30	unknown	regulates plasma membrane H^+^–ATPase [33]	32/29
–	Hsp40	–	rescues previously aggregated proteins [34]	–
–	Hsp42	–	reorganizes cytoskeleton after heat shock [35]	–

sHsps were identified for *C. albicans* and *S. cerevisiae* using the *Candida* Genome database (CGD, www.candidagenome.org) and the *Saccharomyces* Genome database (SGD, www.yeastgenome.org), respectively. The term “Hsp” was used as search criterion. Homologies were determined for amino acid sequences using the ClustalW2 sequence alignment program (www.ebi.ac.uk/Tools/msa/clustalw2/).

In fact, *C. albicans* is one of the leading causes of fungal infections in humans. In healthy persons this fungus occurs as a relatively harmless cohabitant of the normal microflora where it exhibits a commensal lifestyle. Within the body, *C. albicans* is primarily found in the oral cavity, the gastrointestinal and urogenital tract [36], [37]. Certain underlying conditions, however, can result in the transition of *C. albicans* to a pathogenic phase, causing infections which range from superficial infections of the skin or mucosa to life-threatening systemic infections [38]. Patients suffering from HIV or AIDS often develop recalcitrant *C. albicans* infections of the oral mucosa [39]. Besides oral candidiasis, *C. albicans* also causes systemic infections with a crude mortality of approximately 37% [40]. Furthermore, the fungus poses a major problem as the causative agent of vulvovaginal infections. It is estimated that approximately 75% of all women suffer at least once in their lifetime from such infections with approximately 5% experiencing recurrent infections [41], [42]. *C. albicans* possesses an armamentarium of pathogenicity determinants which enable it to cause these infections. Key factors include the yeast-to-hyphal transition [43], the production of adhesins [44] and invasins [22], [45] and the secretion of aspartic proteases [46].

In addition, fitness attributes [47], including metabolic adaptation and flexibility [48], as well as adaptation to different environmental stresses, are also vital for *C. albicans* virulence [49], [50].

In this study we report the identification of a novel sHsp in *C. albicans*. Due to its predicted molecular weight we named the corresponding gene *HSP21* (orf19.822; NCBI-ID: 3637364). By molecular analysis, we demonstrated that this novel sHsp is involved in *C. albicans* adaptation to specific environmental stresses, homeostasis of intracellular stress protectants, immune evasion, as well as pathogenicity. This work represents the first detailed description of a small heat shock protein in *C. albicans* and is the first demonstration of a small heat shock protein contributing to the virulence of a eukaryotic pathogen.

## Results

### *C. Albicans* orf19.822 Encodes a Predicted Small Heat Shock Protein

**Figure 1:**
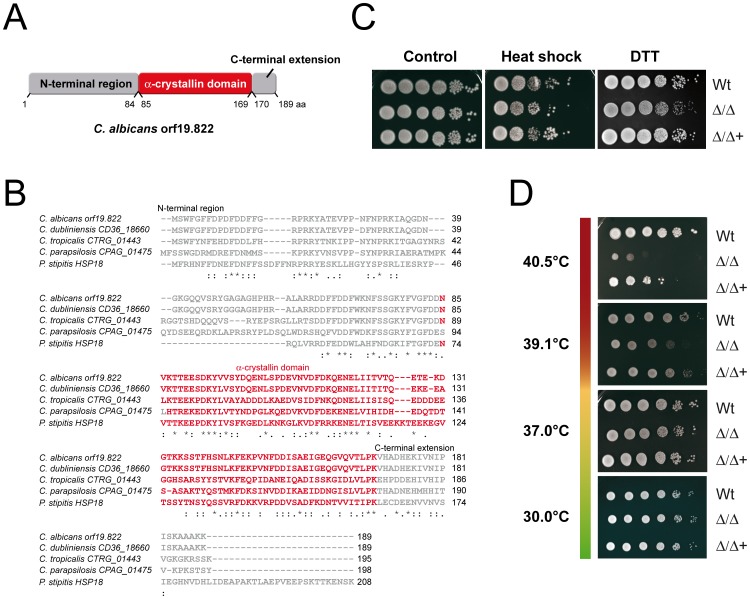
*C. albicans* orf19.822 encodes a predicted sHsp required for adaptation to long-term thermal stress. (A) Structural organization of orf19.822 with a conserved central α-crystallin domain (red) flanked by variable N- and C-terminal domains (grey), based on results from http://www.expasy.ch/prosite/database. Numbers below the structural elements represent amino acid position. (B) Alignment of the orf19.822 protein sequence with orthologues from other organisms (generated with ClustalW2). The conserved α-crystallin-domain sequence is shown in red characters. Identical residues are marked with (*), residues with the same size and hydropathy are marked by (:), residues with the same size or hydropathy are marked by (.). (C) Short-term heat shock and endoplasmic reticulum (ER)-stress. Cells of YPD-overnight cultures of the wild type (Wt), *hsp21*Δ/Δ mutant (Δ/Δ) and *hsp21*Δ/Δ::*HSP21* complemented mutant (Δ/Δ+) were serially diluted from 10^6^ to 10^1^ cells (left to right), either exposed to heat shock (50°C, 15 min) or not (control), plated on YPD and incubated for 2 days at 37°C. ER-stress was induced by growing the cells on YPD agar plates supplemented with 30 mM dithiothreitol (DTT). (D) Growth of the Wt, *hsp21*Δ/Δ mutant (Δ/Δ) and *hsp21*Δ/Δ::*HSP21* complemented mutant (Δ/Δ+) on solid SD minimal medium at temperatures ranging from 30°C to 40.5°C.

orf19.822 was first identified and chosen for detailed investigation according to two criteria. Firstly, orf19.822 was found to be amongst the most strongly upregulated genes in multiple transcriptional profiles of different *C. albicans* infection models as well as in transcriptional profiles for *C. albicans* subjected to different stress conditions (Table S1). Upregulation of orf19.822 was detected during *ex vivo* liver infection (up to 20-fold) [51], interaction with whole blood (up to 4.8-fold) as well as interaction with neutrophils (up to 6.2-fold) [52]. Furthermore, the gene was found to be upregulated under mild oxidative stress (up to 3.2-fold) [53], during interaction with macrophages (up to 29.6-fold) [54] and upon weak acid induced stress (up to 88.7-fold) [55]. Finally, orf19.822 was shown to be highly expressed during heat shock, induced by a shift from either 23–37°C (up to 10.9-fold) [53], 30–42°C (up to 19-fold) [56] or 30–45°C (up to 25-fold) [12]. Secondly, to the best of our knowledge, this gene was of completely unknown function prior to our investigations and a preliminary *in silico* analysis of the protein sequence identified interesting structural features (see below). Using ExPASy PROSITE, we identified a sHsp-typical α-crystallin domain and N- and C-terminal regions within the deduced amino acid sequence of orf19.822 (Figure 1A). A BLASTp search analysis of the amino acid sequence revealed sequence similarities to proteins of unknown function in *Candida dubliniensis* (96% identity), *Candida tropicalis* (51%) and *Candida parapsilosis* (40%). *C. albicans* orf19.822 also displayed significant sequence similarity to the *Pichia stipitis* small heat shock protein Hsp18 (39% identity over the full length of these proteins) (Figures 1B and S1A). The similarity was higher within the α-crystallin domains (42%). No orf19.822 orthologues were identified in the non-pathogenic yeast *Saccharomyces cerevisiae* or, indeed, in any other species outside the CUG clade of fungi [57]. Analysis of the orf19.822 promoter region revealed the presence of two characteristic Hsp heat shock elements (HSEs) [11], [58] as well as one non-standard HSE (nHSE) motif [59]. Furthermore, a Hsp-typical stress-responsive element (STRE) [60], [61], [62] was detected within the orf19.822 promoter region (Figure S1B). Together with the presence of the sHsp-family-defining α-crystallin domain within the amino acid sequence, its transcriptional upregulation under thermal stress, the occurrence of HSEs and STRE in the promoter region, the significant homology to *P. stipitis* Hsp18 and the predicted molecular mass of 21.487 kDa, we refer to *C. albicans* orf19.822 as Hsp21 (heat shock protein 21). Next, we were interested in elucidating whether *HSP21* does indeed play a role in stress adaptation in *C. albicans*. For this purpose we constructed a *hsp21*Δ/Δ homozygous deletion mutant (Figure S2).

### Hsp21 Mediates Thermotolerance and Adaptation to Oxidative Stress in *C. albicans*

Heat shock is known to provoke protein unfolding, disruption of the cytoskeleton, loss of correct organelle localization and intracellular transport breakdown, along with a multitude of other detrimental effects [3]. To prevent and counteract these processes, Hsps are expressed, which protect the cell by acting as molecular chaperones and preventing non-specific protein aggregation. As part of the heat shock response, cells also express sHsps, which efficiently counteract protein aggregation by binding proteins in a sponge-like manner and either directing them to the major Hsps for refolding or to the degradation machinery for disposal [63]. Deletion of the unrelated *HSP12* in *S. cerevisiae* has been shown to result in strongly increased sensitivity of the mutant to heat shock [25]. We therefore first examined the effect of a 15 min heat shock at 50°C on survival of the *C. albicans hsp21*Δ/Δ null mutant strain. In contrast to the *S. cerevisiae hsp12*? mutant [25], deletion of *HSP21* in *C. albicans* only led to a moderately increased sensitivity to heat shock in comparison to the parental wild type and a *hsp21*Δ/Δ::*HSP21* complemented strain (Figure 1C). This indicates that Hsp21 plays a minor role in adaptation to acute, short-term elevations in temperature, but that its function can be largely compensated for either by other Hsps or as yet unidentified sHsps. Analogous to heat shock, the unfolded protein response (UPR) occurs upon endoplasmic reticulum (ER)-stress [64], [65]. Human Hsp90 has been shown to modulate the UPR by stabilizing transmembrane sensor kinases in the ER [66]. To determine whether Hsp21 is involved in the UPR in *C. albicans*, the *hsp21*Δ/Δ mutant was incubated in the presence of dithiothreitol (DTT), an agent that unfolds proteins by reducing disulfide bonds and thereby elicits UPR. Growth of the *hsp21*Δ/Δ mutant was mildly inhibited under ER-stress in comparison to the wild type and the *hsp21*Δ/Δ::*HSP21* complemented strain (Figure 1C), indicating that Hsp21 also plays a minor role in the UPR in *C. albicans*.

**Figure 2:**
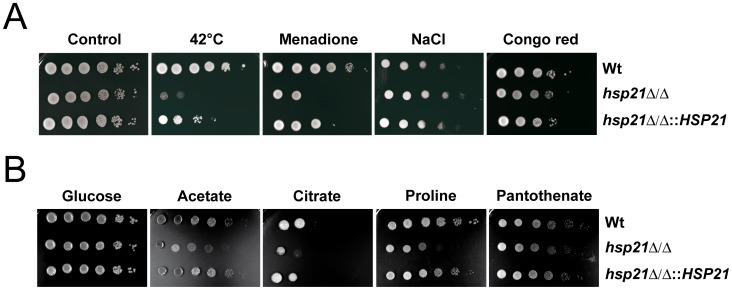
*hsp21*Δ/Δ has increased susceptibility to thermal and oxidative stress and has a growth defect under nutrient limitation. Drop test analysis with serial dilutions of *C. albicans* wild type (Wt), *hsp21*Δ/Δ mutant and *hsp21*Δ/Δ::*HSP21* complemented mutant on agar containing different stressors. (A) Growth of the *hsp21*Δ/Δ mutant on solid SD minimal medium under different environmental stresses, including thermal stress (42°C), oxidative stress (0.4 mM menadione), osmotic stress (1.5 M NaCl) and cell wall stress (450 µg ml^-1^ Congo red). Plates subjected to thermal stress were incubated for 4–5 days, cells grown under non-stress (control), oxidative, osmotic or cell wall stress for 2–3 days at 37°C. Experiments were repeated at least twice yielding similar results. Representative pictures are shown. (B) Drop test analysis with serial dilutions of the indicated strains on agar containing different compounds as sole carbon and nitrogen sources. Agar containing 0.67% yeast nitrogen base plus ammonium sulphate without amino acids was supplemented with 2% glucose, potassium acetate or citrate as sole carbon source. Yeast nitrogen base agar without ammonium sulphate and amino acids was supplemented with 100 µg ml^-1^ proline or pantothenate as sole carbon and nitrogen source. Plates were incubated at 37°C for 3–7 days depending on the carbon and nitrogen source. Experiments were repeated at least twice yielding similar results. Representative pictures are shown.

Certain HSPs, such as *S. cerevisiae HSP70*, are dispensable for surviving short lived exposure to very high temperature, but required for long-term growth under less severe thermal stress [2]. We therefore next examined the role of *CaHSP21* in adaptation to prolonged thermal stress. Strikingly, under constant elevated temperature of 39.1°C the *hsp21*Δ/Δ mutant showed a growth defect, was strongly impaired in growth at a constant temperature of 40.5°C (Figure 1D), and completely unable to grow at 42°C (Figure 2A). Growth was restored by complementation of *hsp21*Δ/Δ with a single copy of *HSP21*, albeit not to wild type levels. The phenotypes of the *hsp21*Δ/Δ mutant – surviving short term exposure to very high temperature, but failing to grow over prolonged periods of thermal stress – is reminiscent of *S. cerevisiae hsp70*? [2]. Hsps and sHsps not only function in adaptation to heat stress but also to other stresses, such as oxidative, osmotic and cell wall stresses. We therefore investigated growth of the *hsp21*Δ/Δ mutant under these environmental stresses. Oxidative stress, induced by menadione - a naphthoquinone which exerts its toxic function mainly through the generation of reactive oxygen species (ROS) [67], [68] – led to a severe growth defect of the *hsp21*Δ/Δ mutant (Figure 2A). This points to a possible role for Hsp21 in preventing non-specific protein aggregation upon exposure of *C. albicans* to ROS. Interestingly, for osmotic stress induced by high concentrations of NaCl, the *hsp21*Δ/Δ mutant was found to be slightly more resistant than the wild type and *hsp21*Δ/Δ::*HSP21* complemented strain. Cell wall directed stress elicited by Congo red – a compound which binds nascent chitin chains and thereby inhibits connection of chitin to β-1,3-glucan and β-1,6-glucan [69] – did not affect growth of the *hsp21*Δ/Δ mutant.

In summary, Hsp21 contributes to adaptation to thermal and oxidative stress, but not to osmotic or cell wall stress and plays only a minor role in the UPR. In addition to these environmental stresses, it has been proposed that nutrient limitation represents a significant stress *in vivo*. We therefore next investigated growth of the *hsp21*Δ/Δ mutant under nutrient-restricted conditions.

### Hsp21 Contributes to Growth Under Conditions of Nutrient Limitation

*In vivo*, *C. albicans* faces a nutrient-limited environment [70]. Moreover, it has been shown that the glyoxylate cycle is required for normal fungal virulence [71]. We therefore cultivated *hsp21*Δ/Δ mutant cells on minimal media supplemented with different carbon (C-) and nitrogen (N-) sources (Figure 2B). The *hsp21*Δ/Δ mutant had a moderate growth defect on media containing acetate or citrate as sole C-source in comparison to the wild type and *hsp21*Δ/Δ::*HSP21* complemented strain. On media containing proline as sole C- and N-source, however, the *hsp21*Δ/Δ mutant displayed a strong growth defect in comparison to the wild type and the *hsp21*Δ/Δ::*HSP21* complemented strain. Similarly, a moderate reduction in growth of the *hsp21*Δ/Δ mutant was observed when pantothenate (vitamin B_5_) was used as sole C- and N- source. These findings indicate that *HSP21* plays an important role in adaptation to nutrient limited conditions, which might be of importance during *in vivo* infections.

### Simultaneous Stresses: Osmotic Stress Bypasses Hsp21-dependent Thermal Stress Tolerance

**Figure 3:**
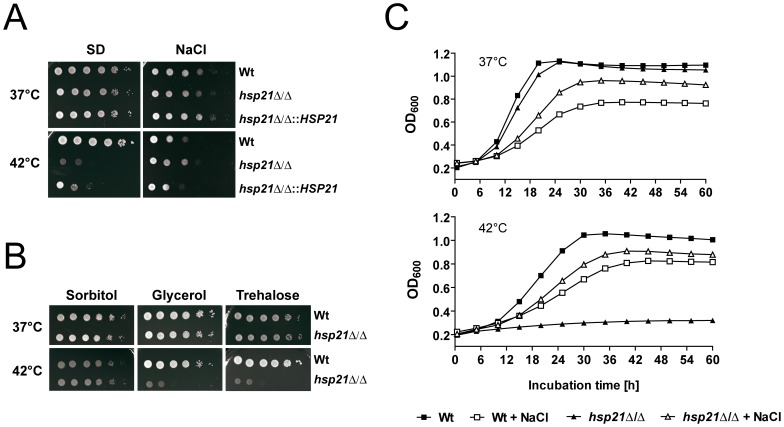
Osmotic stress bypasses the Hsp21-dependent thermal stress tolerance. Simultaneous osmotic and thermal stress lead to growth of the heat-sensitive *hsp21*Δ/Δ mutant. (A) Drop test analysis with serial dilutions of *C. albicans* wild type (Wt), *hsp21*Δ/Δ mutant and *hsp21*Δ/Δ::*HSP21* complemented mutant on SD agar or SD agar containing 1.5 M NaCl. Plates were incubated at 37°C or 42°C. Experiments were repeated at least twice yielding similar results. Representative pictures are shown. (B) Drop test analysis with serial 10-fold dilutions of the wild type and *hsp21*Δ/Δ mutant on SD agar containing 1.5 M sorbitol, 2% glycerol, or 30 mM trehalose. Plates were incubated at 37°C or 42°C. Experiments were repeated twice yielding similar results. Representative pictures are shown. (C) Growth curves for the wild type and *hsp21*Δ/Δ mutant in SD medium and SD medium supplemented with 1.5 M NaCl at 37°C or 42°C. Experiments were repeated twice yielding similar results. Results are the mean of two measurements per strain and time point. Representative growth curves are shown.

During growth within a host, *C. albicans* must adapt to a variety of stresses and it is likely that some of these stresses occur simultaneously. We therefore sought to characterize the role of *HSP21* in adaptation to multiple stresses. The *hsp21*Δ/Δ mutant grew normally at 37°C on solid SD medium, exhibited wild type resistance to osmotic stress and did not grow at 42°C as described above (Figure 3A). Strikingly, the combination of NaCl-induced osmotic stress and thermal stress fully bypassed Hsp21-dependence for growth under thermal stress (Figure 3A). We observed the same phenomenon for growth under thermal stress combined with sorbitol- and potassium chloride-induced osmotic stress (Figure 3B and data not shown). Osmotic stress is known to elicit a protective intracellular glycerol accumulation. It has been shown for *Escherichia coli* that osmolytes such as glycerol and trehalose not only stabilize the medium under osmotic stress, but, importantly, also can act as chemical chaperones by stabilizing native proteins, preventing protein aggregation and helping in refolding unfolded polypeptides under thermal stress [72], [73]. We therefore also tested growth of the mutant at 42°C in the presence of exogenously added glycerol or trehalose, however, this did not restore growth of the *hsp21*Δ/Δ mutant (Figure 3B).

In liquid SD medium at 37°C, both wild type and *hsp21*Δ/Δ grew at similar rates. Addition of NaCl to the medium inhibited growth of the wild type; interestingly, under this osmotic stress condition, deletion of *HSP21* increased the growth rate relative to the wild type (Figure 3C upper panel). Under thermal stress (42°C) the *hsp21*Δ/Δ mutant failed to grow. However, under simultaneous thermal and osmotic stress, growth of the *hsp21*Δ/Δ mutant surpassed that of the wild type (Fig. 3C lower panel). We reasoned that the growth defect of the *hsp21*Δ/Δ at elevated temperature may be due to heat-induced lysis of the mutant cells, and that the addition of exogenous osmolytes simply stabilized the cell membrane. We therefore incubated wild type and mutant at 42°C in SD medium for 5 h and measured cellular viability with methyl blue staining. Both strains exhibited similar low levels (∼10%) of inviable cells. Therefore the thermal stress growth defect of *hsp21*Δ/Δ was not due to temperature-induced cellular lysis, indicating that the observed osmolyte-rescue was unlikely to be solely the result of membrane stabilization under this experimental setting.

### Hsp21 Contributes to Hyphal Formation and is Required for Invasive Hyphal Growth of *C. albicans*

**Figure 4:**
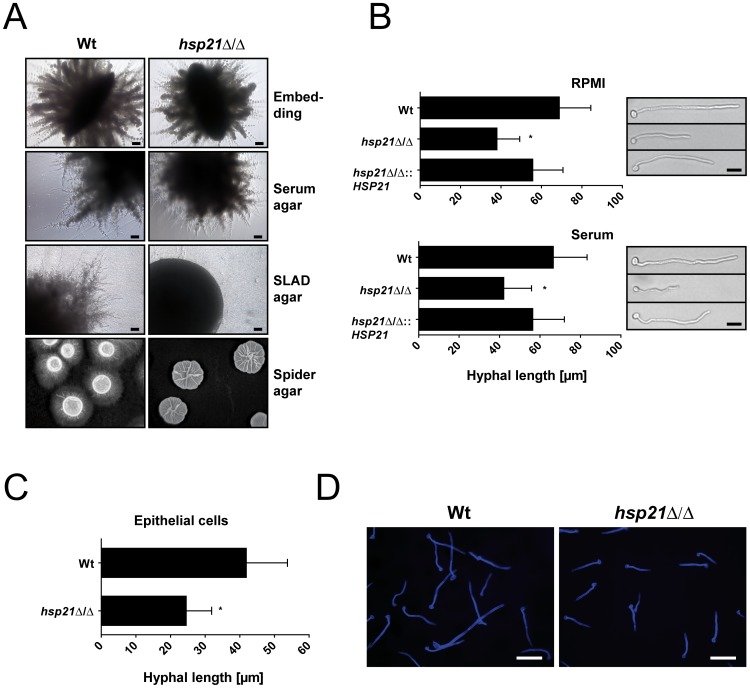
*hsp21*Δ/Δ exhibits reduced invasive growth and hyphal formation. (A) Formation of hyphae was induced by embedding fungal cells in YPS (2% saccharose) agar or by plating them on solid water agar supplemented with 10% fetal bovine serum, SLAD agar or on solid Spider medium. Serum agar plates were incubated for 2, SLAD agar plates for 4, and Spider agar plates for 10 days at 37°C. Embedded plates were incubated at 25°C for 5 days. Experiments were performed twice in duplicate. Representative pictures are shown. Scale bar: 100 µm. (B) Hyphal elongation in RPMI1640 and 10% serum. Wild type, *hsp21*Δ/Δ mutant or *hsp21*Δ/Δ::*HSP21* complemented mutant cells were grown overnight in SD medium. After washing twice with water, 10^4^ cells were incubated in RPMI1640 or water supplemented with 10% serum in 24-well cell culture plates at 37°C for 4 hours in the presence of 5% CO_2_. Hyphal lengths were then determined using an Inverse microscope (Leica). Results are the mean ± SD of two independent experiments, each performed in duplicate with the length of at least 100 cells measured per strain and experiment. *P<0.0001 compared with the wild type and *hsp21*Δ/Δ::*HSP21* complemented strain. Pictures of representative hyphae were taken using a 40x-magnification. Scale bar: 10 µm. (C) Hyphal formation on epithelial monolayers. TR146 epithelial cells were cultured to confluency and infected with *C. albicans* cells for three hours. Fungal cells were then stained with Calcofluor white (stains invaded and non-invaded fungal elements) and hyphal lengths were determined by fluorescence microscopy. Results are the mean ± SD of two independent experiments, each performed in duplicate with the length of at least 200 cells measured per strain and experiment. *P<0.0001 compared with the wild type strain. (D) Representative pictures of wild type and *hsp21*Δ/Δ hyphae are shown. Scale bar: 20 µm.

It has recently been shown by Cowen and colleagues that the molecular chaperone and heat-shock protein Hsp90 acts as physiological link between fungal morphogenesis and temperature [74], [75]. We therefore postulated that Hsp21 may also play a role in morphogenesis. Hyphal formation was induced by embedding fungal cells in yeast peptone saccharose agar, by plating cells on agar supplemented with 10% fetal bovine serum, on SLAD agar or on spider medium agar [76] (Figure 4A). The *hsp21*Δ/Δ mutant cells formed filamentous colonies under embedded conditions and on serum-containing agar, however, colonies appeared to be smaller than those of the wild type. The reduced colony size appeared to be mainly due to shorter radial filaments produced by the *hsp21*Δ/Δ mutant in comparison to the wild type. An even more striking phenotype caused by deletion of *HSP21* was observed on SLAD and Spider agar. On SLAD agar the *hsp21*Δ/Δ mutant formed aberrant colonies which completely lacked the peripheral filaments observed for the wild type. When grown on Spider agar the wild type typically forms colonies with a central wrinkled area consisting of yeast, hyphae and pseudohyphae and a peripheral area consisting mainly of agar-invading filaments [77]. In contrast, the *hsp21*Δ/Δ mutant developed wrinkled colonies that completely lacked peripheral hyphae. We conclude that *C. albicans HSP21* is required for optimal invasive growth in agar. In order to further characterize the hyphal formation defect of the *hsp21*Δ/Δ mutant, we next investigated filamentation on a single cell level in liquid hyphae inducing media (Figure 4B). While wild type and revertant cells formed hyphae with a mean length of around 60 µm, the *hsp21*Δ/Δ mutant filaments only reached a mean length of around 40 µm upon exposure to RPMI or 10% serum for 4 h at 37°C and 5% CO_2_. In order to more closely mimic an *in vivo* situation, we also induced hyphal formation by incubation on oral epithelial cells for 3 h at 37°C and 5% CO_2_ (Figure 4C and 4D). Here, wild type cells reached a length of about 40 µm. Again, *hsp21*Δ/Δ hyphae were significantly shorter, reaching only about 25 µm. Taken together, these results indicate that Hsp21 contributes to hyphal formation in *C. albicans*. Together with the prominent stress phenotypes of the *hsp21*Δ/Δ mutant (above) we concluded that *HSP21* represents a promising virulence factor candidate and continued by investigating the role of *HSP21* during infection.

### A *hsp21*Δ/Δ Mutant is More Susceptible to Killing by Human Phagocytes

**Figure 5:**
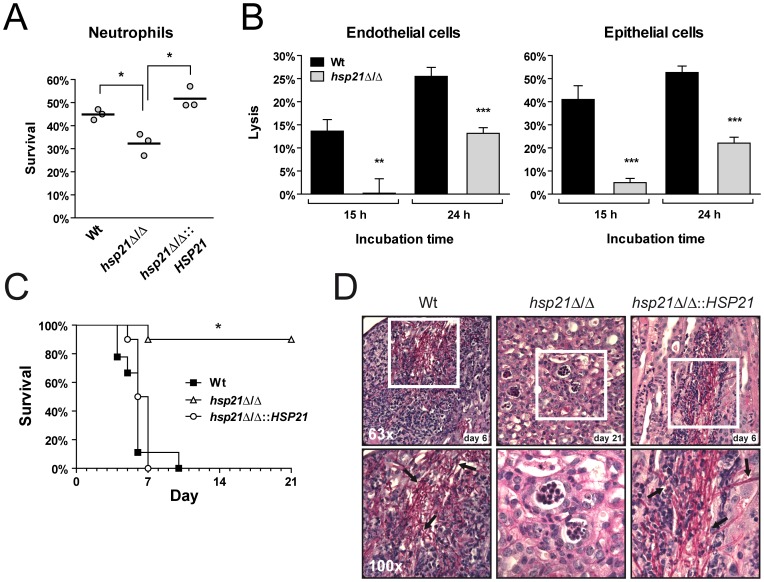
Hsp21 is a virulence factor. (A) The deletion of *HSP21* leads to increased susceptibility of *C. albicans* to killing by human neutrophils. Wild type (Wt), *hsp21*Δ/Δ mutant and *hsp21*Δ/Δ::*HSP21* complemented mutant cells were exposed to human neutrophils for three hours and viability was then determined by plating on YPD agar. Experiments were performed three times. The bar represents the mean of these single values. *P<0.01 compared with the wild type and *hsp21*Δ/Δ::*HSP21* complemented strain. (B) Hsp21 is required for *C. albicans* to cause full damage to endothelial and oral epithelial cells *in vitro*. Monolayers of human-derived endothelial and oral epithelial cells were infected with *C. albicans* wild type (Wt) and *hsp21*Δ/Δ mutant strains for 15 or 24 h. Host cell damage was then determined by measuring lactate dehydrogenase (LDH) levels. Results are the mean ± SD of at least three independent experiments, each performed in triplicate. **P<0.01 and ***P<0.001 compared with the wild type strain. (C) The *hsp21*Δ/Δ mutant is avirulent in a mouse model of hematogenously disseminated candidiasis. Female Balb/C mice (n = 10 mice per *C. albicans* strain) were challenged intravenously with either the wild type (Wt), the *hsp21*Δ/Δ mutant or the *hsp21*Δ/Δ::*HSP21* complemented strain via the lateral tail vein. *P<0.0001 compared with mice either infected with the wild type or *hsp21*Δ/Δ::*HSP21* complemented strain. (D) Periodic acid Schiff staining of kidney sections from mice infected with the wild type and *hsp21*Δ/Δ::*HSP21* complemented strain six days, and with the *hsp21*Δ/Δ mutant strain 21 days post infection. Pictures were taken at 63x (upper panel) and 100x magnification (lower panel). The lower panel of images show magnifications of the white boxed areas from the above images. Arrows point to *C. albicans* filaments within the tissue.

Attack by phagocytic cells of the innate immune system represents a significant stress to invading microorganisms. Given the severe stress adaptation defects observed upon *HSP21* deletion (above), and the strong transcriptional upregulation of *HSP21* upon exposure to both macrophages and neutrophils (Table S1), we hypothesized that Hsp21 may play a role in defending fungal cells from attack by phagocytes. We initially tested the survival of wild type and *hsp21*Δ/Δ mutant cells following exposure to macrophages derived from the immortalized monocyte cell line, THP1. Overall, fungal killing by THP1 cells was low (10–30%), and although we observed a modest decrease in survival of the *hsp21*Δ/Δ mutant, this difference was not significant (data not shown). Neutrophils play a crucial role in controlling *C. albicans* infections [52], [78], [79]. We therefore investigated the survival of *C. albicans* following a 3 h co-incubation with human neutrophils. As shown in Figure 5A, deletion of *HSP21* significantly reduced survival from 45% (wild type) to 32%; complementation of the mutant with a single copy of *HSP21* significantly restored survival to 51%.

Therefore, Hsp21 is not only required for resisting certain *in vitro* stresses (above), but also plays a role in surviving attack by neutrophils.

### Hsp21 is Involved in Damage of Human-derived Endothelial and Oral Epithelial Cells *in Vitro*

The morphological and stress defects of *hsp21*Δ/Δ (above), together with the transcriptional upregulation of *HSP21* during various models of infection, suggested that Hsp21 may play a role in fungal pathogenesis. In order to investigate the role of Hsp21 during host-pathogen interactions, we used a lactate dehydrogenase colorimetric assay to determine damage to monolayers of endothelial and epithelial cells caused by the different *C. albicans* strains [80], [81]. Interestingly, the *hsp21*Δ/Δ mutant caused significantly reduced damage of both cell lines after 15 and 24 hours of infection (Figure 5B). The mutant caused 98% less damage to endothelial cells in comparison to the wild type after 15 hours and 48% less damage after 24 hours post infection. A similarly strong reduction was observed with epithelial cells. Eighty eight percent less epithelial destruction was determined for the mutant after 15 hours and 58% less damage after 24 hours, compared to the wild type. Complementation of the *hsp21*Δ/Δ mutant with *HSP21* restored *C. albicans* capacity to damage monolayers of epithelial cells (Figure S3). *C. albicans* adherence to and invasion of host cells is a prerequisite for host cell damage [82]. We therefore tested the endothelial adhesion and epithelial invasion capacities of *hsp21*Δ/Δ, however, adhesion and invasion levels were comparable to the wild type (Figure S4), suggesting that the strong reduction in damage was not due to decreased adhesion or invasion.

### Hsp21 Promotes Virulence of *C. Albicans*

The morphological and stress defects, reduced pathogenicity in *in vitro* infection models and impaired survival upon co-incubation with neutrophils of *hsp21*Δ/Δ, suggested that Hsp21 represents a promising candidate virulence factor.

We therefore next investigated the impact of *HSP21* deletion on *C. albicans* virulence *in vivo*. For this purpose we first used an alternative embryonated hen egg infection model of candidiasis [83], [84].

Within the first day of infection, the percentages of embryos killed by the wild type (55%) and mutant (40%) were very similar, but then subsequently diverged during the course of the ongoing infection: while wild type infected embryos continued to rapidly succumb to infection, few *hsp21*Δ/Δ-infected embryos died after two days post-infection (Figure S5). By the end of the experiment (seven days post-infection), only 10% of embryos infected with the wild type were still alive, while 35% of embryos infected with the mutant survived. Hence, Hsp21 appears to play a major role in the later stages of *in ovo* infection.

To characterize the role of Hsp21 during mammalian infection, we used a murine model of hematogenously disseminated candidiasis (Figure 5C). Survival of mice infected with the wild type or the *hsp21*Δ/Δ::*HSP21-*reconstituted strain showed no significant difference. All mice infected with these strains had to be euthanized within seven to ten days post infection. In contrast only one (of the ten) mice infected with the *hsp21*Δ/Δ mutant had to be sacrificed seven days post infection. All remaining mice survived until the end of the experiment, 21 days post infection. Kidney fungal burdens of (*hsp21*Δ/Δ-infected) end-point surviving mice was found to be 1×10^4^ CFU per gram, indicating that very few *hsp21*Δ/Δ cells remained viable in the kidneys of infected mice.

Histological examination of kidneys infected with the *hsp21*Δ/Δ mutant revealed far fewer fungal cells in comparison to the wild type and *hsp21*Δ/Δ::*HSP21-*reconstituted strain (Figure 5D). Also, extensive neutrophil infiltration at fungal foci (consisting of hyphae and/or pseudohyphae) was observed for the wild type and *hsp21*Δ/Δ::*HSP21-*complemented strain, whilst kidneys infected with the *hsp21*Δ/Δ mutant displayed a homogenous neutrophil distribution. This data suggests that *hsp21*Δ/Δ did not elicit significant inflammation within the kidney.

### Hsp21 Regulates Homeostasis of Intracellular Stress-protective Molecules

Having demonstrated that *HSP21* is involved in both adaptation to stress and virulence, we next sought to determine the cellular mechanisms by which Hsp21 protects *C. albicans* from stressful environments. Stressful conditions not only induce the heat shock response with the expression of Hsps and sHsps, but also the synthesis of stress-protective molecules. These compounds play crucial roles in cellular protection, ranging from stabilizing osmotic and ionic misbalances, nutrient storage to stabilizing stress-labile proteins in a chaperone-like manner. The major stress-protectant molecules are glycerol, glycogen and trehalose, all three of which are synthesized from intermediates of glycolysis [85], [86]. Given the severe growth defects of *hsp21*Δ/Δ under thermal and oxidative stress, we hypothesized that Hsp21 may regulate the homeostasis of stress protective molecules. We therefore systematically analyzed the intracellular levels of all three stress protective molecules during growth under a range of stresses.

**Figure 6:**
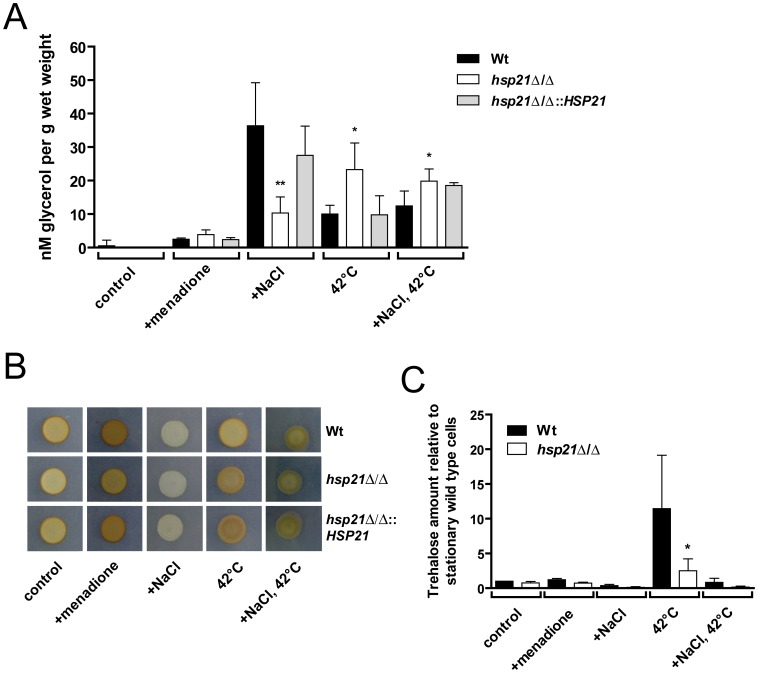
Hsp21 regulates intracellular glycerol, glycogen and trehalose homeostasis. (A) Measurement of intracellular glycerol levels in the wild type (Wt), the *hsp21*Δ/Δ mutant or the *hsp21*Δ/Δ::*HSP21* complemented strain after growth for 24 h in SD medium (control) at 30°C, SD medium supplemented with 0.4 mM menadione (+menadione) at 30°C, SD medium supplemented with 1.5 M NaCl (+NaCl) at 30°C, SD medium at 42°C (42°C), or SD medium supplemented with 1.5 M NaCl at 42°C (+NaCl, 42°C). Glycerol levels are plotted in nM normalized against wet weight (g). Results are the mean ± SD of three independent experiments. **P<0.01 and *P<0.05 compared with the wild type and *hsp21*Δ/Δ::*HSP21* complemented strain. (B) Estimation of glycogen content with iodine vapour for the wild type (Wt), the *hsp21*Δ/Δ mutant or the *hsp21*Δ/Δ::*HSP21* complemented strain after cultivation on SD agar (control) at 37°C, SD agar supplemented with 0.4 mM menadione at 37°C (+menadione), SD agar supplemented with 1.5 M NaCl (+NaCl) at 37°C, SD agar at 42°C (42°C), or SD agar supplemented with 1.5 M NaCl at 42°C (+NaCl, 42°C). The darker the colour of a colony, the more intracellular glycogen is present. Experiments were performed twice in duplicate yielding similar results. Representative pictures are shown. (C) Measurement of intracellular trehalose levels in the wild type (Wt) and the *hsp21*Δ/Δ mutant strain. Growth conditions were the same as described for panel (A). Trehalose levels (nmol trehalose per mg total cell protein) are indicated relative to the Wt grown under control conditions. Results are the mean ± SD of five (control; +NaCl; 42°C) or two (+menadione; +NaCl, 42°C) independent experiments. *P<0.05 compared with the wild type strain under the same condition.

Only very low levels of intracellular glycerol could be measured under non-stress conditions in the wild type (1.6 nM/g wet weight), the *hsp21*Δ/Δ mutant (0.4 nM/g wet weight) and the *hsp21*Δ/Δ::*HSP21-*reconstituted strain (0.2 nM/g wet weight) (Figure 6A). Oxidative stress (0.4 mM menadione) resulted in moderate accumulation of glycerol for the different strains. Osmotic stress, induced by 0.5 M NaCl however, led to a strong accumulation of glycerol in the wild type (36.4 nM/g wet weight). Interestingly, the *hsp21*Δ/Δ mutant (10.3 nM/g wet weight) produced 72% less glycerol than the wild type upon osmotic stress. Complementation of the mutant with *HSP21* restored wild type glycerol production (27.5 nM/g wet weight).

Thermal stress also resulted in glycerol accumulation in wild type cells (10.0 nM/g). Interestingly, the glycerol content of *hsp21*Δ/Δ cells upon thermal stress was over twice that of the wild type (23.3 nM/g wet weight) (Figure 6A). Again, complementation of the mutant with *HSP21* restored wild type glycerol levels (9.8 nM/g wet weight). These results demonstrate that *HSP21* is required for normal glycerol homeostasis under both osmotic and thermal stress conditions in *C. albicans*. Surprisingly, osmotic stress under elevated temperatures did not induce glycerol accumulation by *C. albicans*.

We next investigated cellular glycogen levels under oxidative, osmotic and thermal stress by iodine vapour staining (Figure 6B). Cells that contain high concentrations of glycogen are stained darker upon exposure to iodine vapour [86], [87]. Interestingly, even under control conditions, the *hsp21*Δ/Δ mutant exhibited slightly reduced glycogen content as indicated by lighter iodine staining. Oxidative stress induced the most prominent glycogen accumulation in the wild type. Under this condition, *hsp21*Δ/Δ stained lighter than the wild type. Complementation with *HSP21* restored wild type levels of oxidative stress-induced glycogen. For osmotic stress induced by NaCl, iodine staining was notably lighter than under control conditions and no differences in colour was observed amongst strains. Upon thermal stress, *hsp21*Δ/Δ colonies appeared slightly darker than the wild type, however this was not restored in the *hsp21*Δ/Δ::*HSP21* complemented strain. Finally, the combination of osmotic and thermal stress resulted in darker staining than under osmotic stress alone, and this was independent of *HPS21*. Together these data suggest that *HSP21* is required for the maintenance of normal glycogen levels, primarily under non-stress and oxidative stress conditions.

Finally, we investigated the effects of oxidative, osmotic and thermal stress on intracellular trehalose content (Figure 6C). We observed no increase in trehalose levels for cells exposed to oxidative stress. However, osmotic stress significantly down-regulated trehalose levels in comparison to control cultures. Thermal stress, on the other hand, induced strong trehalose production in the wild type. Strikingly, although *hsp21*Δ/Δ cells responded to thermal stress by accumulating trehalose, levels were more than 4-fold reduced compared to the wild type. Interestingly, the combination of thermal and osmotic stress resulted in a very low trehalose production by the wild type, very similar to that observed under control conditions at 30°C. The *hsp21*Δ/Δ mutant, however, showed an even greater reduction in trehalose levels under the combined stresses in comparison to the control condition. Together our data demonstrate that *C. albicans* responds specifically to different stresses by synthesizing different intracellular stress-protective molecules: osmotic stress induced glycerol synthesis; oxidative stress, glycogen; and thermal stress results in trehalose production. Remarkably, all three stress protectants were mis-regulated in the *hsp21*Δ/Δ mutant under their respective induction conditions. In summary, Hsp21 fine-tunes the cellular balance of the three major stress protectant molecules, depending on the specific nature of the environmental insult.

### Mutations in the Trehalose Pathway Phenocopy *HSP21* Deletion

**Figure 7:**
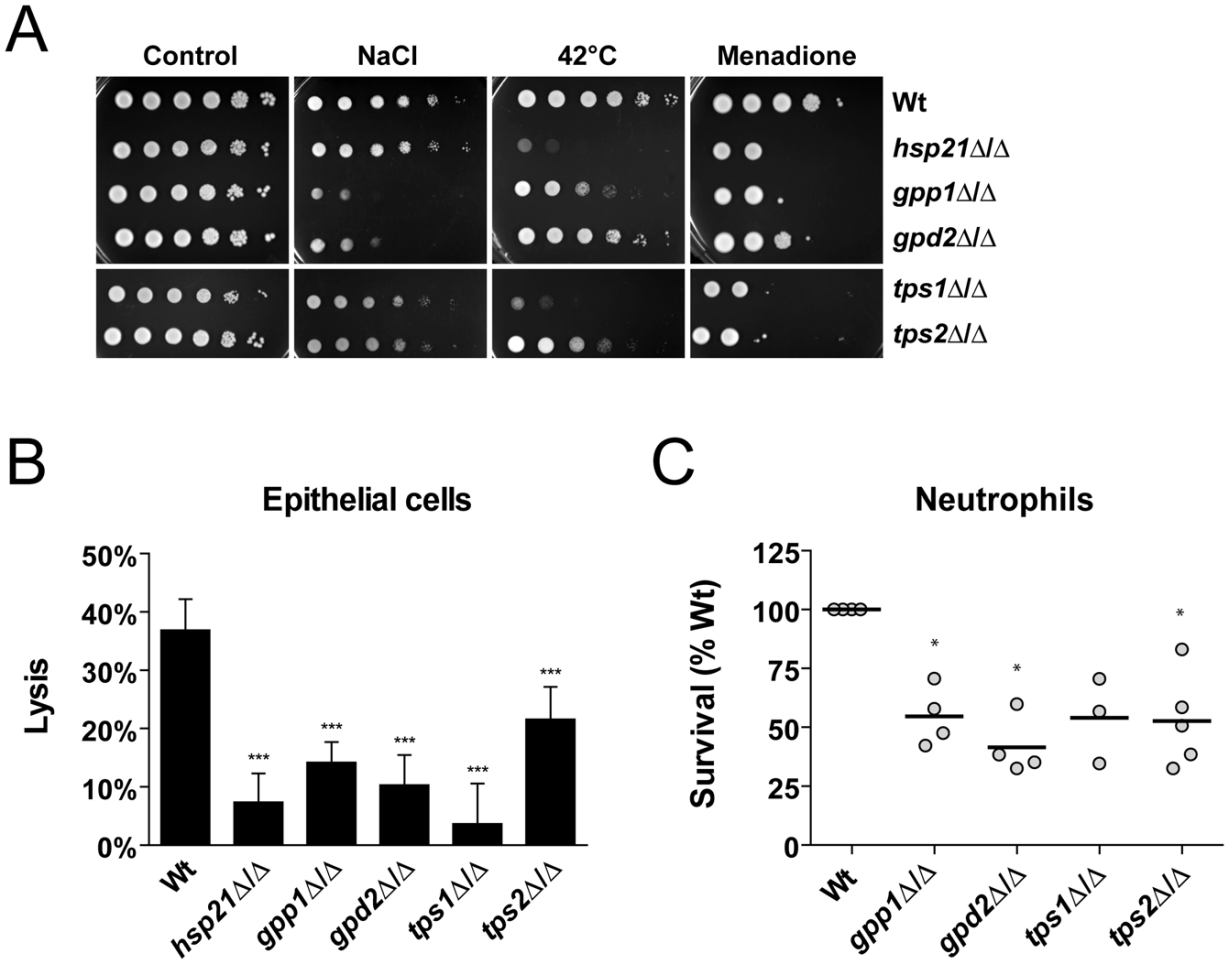
Mutants defective in trehalose synthesis phenocopy *HSP21* deletion. (A) Drop test analysis with serial dilutions of the wild type (Wt) and the indicated mutant strains on SD minimal medium under different environmental stresses, including osmotic stress (1.5 M NaCl), thermal stress (42°C) and oxidative stress (0.4 mM menadione). Plates subjected to thermal stress were incubated for 4–5 days, cells grown under non-stress (control), osmotic or oxidative stress for 2–3 days at 37°C. Experiments were repeated at least twice yielding similar results. Representative pictures are shown. (B) Capacity of the indicated strains to damage oral epithelial cells. Monolayers of epithelial cells were infected with the different strains for 15 h and host cell damage was then quantified by measuring LDH levels. Results are the mean ± SD of two independent experiments, each performed in septuplicate. ***P<0.0001 compared with the wild type strain. (C) Neutrophil killing assay. Cells of the indicated strains were exposed to human neutrophils for three hours and viability was then determined by plating on YPD agar. Wild type survival was set to 100%. BWP17+CIp30 was used as wild type control for *gpp1*Δ/Δ and *gpd2*Δ/Δ, and CAI4+CIp10 was used as wild type control for *tps1*Δ/Δ and *tps2*Δ/Δ. Experiments were performed at least three times. The bar represents the mean of the single values. *P<0.01 compared with the wild type.

In order to confirm the role of Hsp21 in the homeostasis of glycerol and trehalose levels, we next used *C. albicans* mutant strains with deletions in *GPP1* (encoding a putative glycerol 3-phosphatase) [82], *GPD2* (encoding a predicted glycerol 3-phosphate dehydrogenase) [82], *TPS1* (encoding a trehalose-6-phosphate synthase) [88] or *TPS2* (encoding a trehalose-6-phosphate phosphatase) [89]. First, the *gpp1*Δ/Δ, *gpd2*Δ/Δ, *tps1*Δ/Δ and *tps2*Δ/Δ mutants were investigated using drop dilution assays on solid SD minimal medium under osmotic, thermal or oxidative stress (Figure 7A). As previously described, *gpp1*Δ/Δ exhibited a severe growth defect under osmotic stress [90]. *gpd2*Δ/Δ also displayed a strong defect in growth under this condition. The *tps1*Δ/Δ mutant had a severe growth defect at 42°C while *tps2*Δ/Δ and *gpp1*Δ/Δ had a moderate growth defect under elevated temperature. Oxidative stress, elicited by treatment with menadione resulted in impaired growth for all strains.

Second, the capacity of each mutant to damage oral epithelial cells was investigated *in vitro* (Figure 7B). All mutants were significantly attenuated in their damage capacity, with *tps1*Δ/Δ displaying the strongest reduction.

Third, we investigated the interaction of each mutant with human primary neutrophils. Again, all mutants tested showed a similar phenotype as *hsp21*Δ/Δ and were more susceptible to killing activities by these phagocytes (Figure 7C, see also Figure 5A). The *gpp1*Δ/Δ, *gpd2*Δ/Δ and *tps2*Δ/Δ mutants were significantly reduced in their survival by approximately 50%. The *tps1*Δ/Δ mutant had a similarly strong reduction in survival, although not significant. Collectively, these results indicate that mutants defective in trehalose synthesis phenocopy *hsp21*Δ/Δ, and that Hsp21 therefore is likely to operate in or affect this pathway.

### Hsp21 Regulates Cek1-activation under Thermal Stress

Given the role of Hsp21 in governing glycerol, glycogen and trehalose homeostasis and adaptation to both thermal and oxidative stress, we hypothesised that Hsp21 may directly function in one or more of the stress responsive signalling pathways.

**Figure 8:**
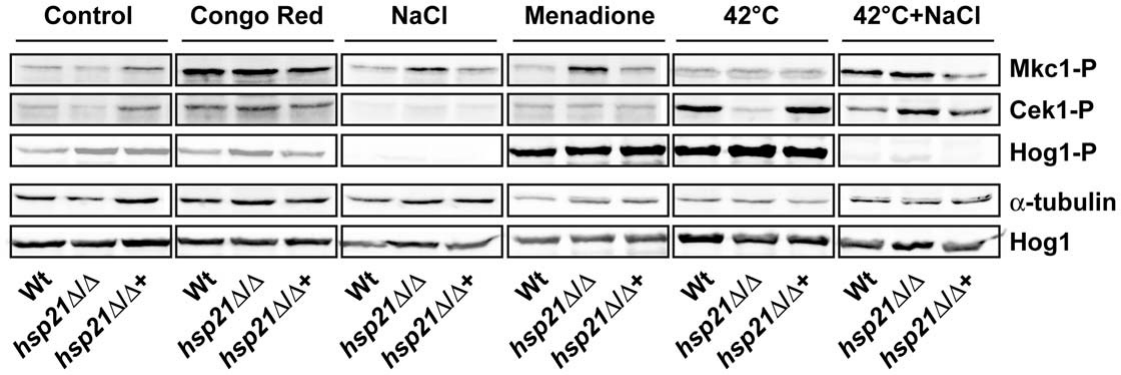
Cek1 phosphorylation in response to thermal stress is Hsp21-dependent. Western blot analysis of phosphorylated Cek1, Mkc1 or Hog1. The wild type (Wt), *hsp21*Δ/Δ mutant and *hsp21*Δ/Δ::*HSP21* complemented strain were incubated under non-stress conditions (control), conditions of cell wall stress (Congo red), osmotic stress (NaCl), oxidative stress (menadione), thermal stress (42°C) or a combination of thermal and osmotic stress (42°C+NaCl) for 4 hours at 30°C or 42°C. Equal amounts of protein extracts were blotted and probed for phosphorylated Cek1 (Cek1-P) and Mkc1 (Mkc1-P). Blots were then stripped and re-probed for α-tubulin (loading control). Hog1 phosphorylation (Hog1-P) was investigated in separate blots and, after stripping, blots were probed for total Hog1 (phosphorylated plus un-phosphorylated) as loading control. Note that thermal stress induces Cek1 phosphorylation in a Hsp21-dependent manner and that simultaneous osmotic stress bypasses Hsp21-dependence.

We therefore undertook a systematic analysis of the three main mitogen-activated protein (MAP) kinase signaling pathways (Mkc1, Cek1 and Hog1) [91], [92] using western blotting in *C. albicans* wild type, *hsp21*Δ/Δ and *hsp21*Δ/Δ+*HSP21* strains. We investigated phosphorylation of Mkc1, Cek1 and Hog1 under five different environmental stress conditions, including cell wall, osmotic, oxidative, thermal, and combined thermal and osmotic stress (Figure 8).

Mkc1 was strongly phosphorylated under cell wall stress (Congo red) but not in response to NaCl, menadione or elevated temperatures; the combination of thermal and osmotic stress, however, resulted in Mkc1 phosphorylation. Interestingly, deletion of *HSP21* rendered Mkc1 phosphorylation responsive to osmotic and oxidative stress.

Cek1 was modestly induced by Congo red and robustly phosphorylated under elevated temperature in *C. albicans*. Importantly, deletion of *HSP21* precluded Cek1 phosphorylation in response to thermal stress. Moreover, the combination of thermal and osmotic stress rescued Cek1 phosphorylation in *hsp21*Δ/Δ (Fig. 8).

Although osmotic stress initially activates the Hog1 pathway [93], at the time point investigated in the current study (4 h), osmotic stress resulted in Hog1 dephosphorylation; whilst oxidative and thermal stress induced robust Hog1 activation. Strikingly, the addition of NaCl to cells grown under thermal stress strongly dephosphorylated Hog1 in comparison to heat stress alone, suggesting that, by 4 h, osmotic stress downregulation of Hog1 bypasses thermal induction.

## Discussion

In this paper, we present the first characterization of a small heat shock protein in *C. albicans* and demonstrate its requirement for intracellular stress protectant homeostasis, environmental stress adaptation, Cek1 phosphorylation, host-pathogen interactions and virulence of this major human fungal pathogen.

As yet, few investigations have focused on the role of small Hsps (sHsps) in microbial pathogenicity. However, it is known that expression levels of sHsps generally increase in response to environmental stresses [3]. Therefore, sHsps may play an important role during microbial infection. Indeed, the novel sHsp encoding *C. albicans* gene orf19.822 (*HSP21*) has been shown to be strongly upregulated under such environmental stress conditions, including thermal, oxidative and acetic acid stress as well as in several models of infection (Table S1). sHsps are defined by a central α-crystallin domain, flanked by a variable C-terminal extension and a non-conserved N-terminal arm and are phylogenetically, structurally and functionally distinct from classical HSPs. *In silico* analysis of the Hsp21 amino acid sequence revealed the presence of such an sHsp-typical core α-crystallin domain, flanked by C- and N-terminal regions. Strengthening this finding, promoter analysis led to the detection of two heat shock elements (HSEs), one non-standard HSE (nHSE) as well as one stress responsive element (STRE). It has recently been shown that transcription of Hsp-encoding genes, such as *HSP70*, *HSP90* and *HSP104*, is regulated by binding of the heat shock transcription factor Hsf1 to HSEs in *C. albicans*, specifically in response to thermal stress. The nHSE, on the other hand, was shown to be non-functional [12]. It is therefore likely that expression of *HSP21* may also be regulated by Hsf1 or other heat shock transcription factors via the HSEs in its promoter. The role of the nHSE remains unclear, although it might be important for *HSP21* expression under stress conditions other than heat shock. During exposure of *C. albicans* wild type cells to weak acid stress *HSP21* is amongst the most strongly induced genes and it has been proposed that *HSP21* expression is regulated by Mnl1 [55]. However, in contrast to Mnl1, Hsp21 was not required for resistance to acetic acid stress (data not shown).

*HSP21* has no orthologue in the non-pathogenic yeast *S. cerevisiae*. Indeed, sequence similarities to Hsp21 on the protein level were detected exclusively for four uncharacterized proteins in fungal species belonging to the CUG clade, which translate this codon to serine instead of leucine. Interestingly, the first three best hits were found in *C. dubliniensis*, *C. tropicalis* and *C. parapsilosis* (Figure 1), which are pathogenic fungi, indicating that Hsp21 orthologues may play a role in the virulence of these non-*albicans* species. The remaining protein, with the lowest homology (Hsp18) belonged to the non-pathogenic yeast *Pichia stipitis*. The relatively low identity of 39% might point to a divergent function in this yeast.

Interestingly, despite robust transcriptional induction of *HSP21* upon heat shock [12], a *hsp21*Δ/Δ mutant displayed only moderate sensitivity to short-term heat shock. This phenomenon is reminiscent of the *S. cerevisiae HSP70* mutant, which has a similar phenotype, i.e. a growth defect at higher temperatures but wild type tolerance to short-termed heat shocks [2], [94], [95]. *C. albicans* Hsp21 might therefore have comparable functions to Hsp70 or cooperate with *C. albicans* Hsp70, for example by transferring partially unfolded client proteins to the Hsp70/Hsp100 disassembling machinery. Such a cooperation has been shown to exist between the *S. cerevisiae* sHsp Hsp26 and the major Hsps Ssa1 (Hsp70) and Hsp104 [96]. As there is no *S. cerevisiae* Hsp26 orthologue in *C. albicans*, it is tempting to speculate that CaHsp21 may have taken over a similar function.

Although relatively tolerant to surviving short term heat shock, *hsp21*Δ/Δ was unable to grow at elevated temperatures. Moreover, *HSP21* contributes to growth under oxidative and nutrient stress, but not osmotic or cell wall stress. Therefore, Hsp21 is specifically required for growth under particular environmental conditions. Heat shock proteins (including sHsps) function by binding to and stabilizing their clients, preventing their unfolding and aggregation [2], [63]. Although further studies are required to reveal the full repertoire of Hsp21 clients, we have identified the mechanistic outcome of *HSP21* deletion: disrupted homeostasis of the three major cellular stress protectant molecules: glycerol, glycogen and trehalose. Osmotic stress induced strong glycerol accumulation, with simultaneous downregulation of glycogen and trehalose levels. Conversely, thermal stress did not effect glycogen levels but stimulated glycerol production and high levels of trehalose accumulation. Only oxidative stress elicited a detectable increase in glycogen levels. Cellular homeostasis of all three molecules was mis-regulated in the *hsp21*Δ/Δ mutant. Osmotic stress resulted in lower glycerol induction than in the wild type, however this defect did not manifest as a higher level phenotype – *hsp21*Δ/Δ grew well under osmotic stress. On the other hand, reduced glycogen levels in *hsp21*Δ/Δ cells under oxidative stress correlates well with heightened sensitivity to this stress.

**Figure 9:**
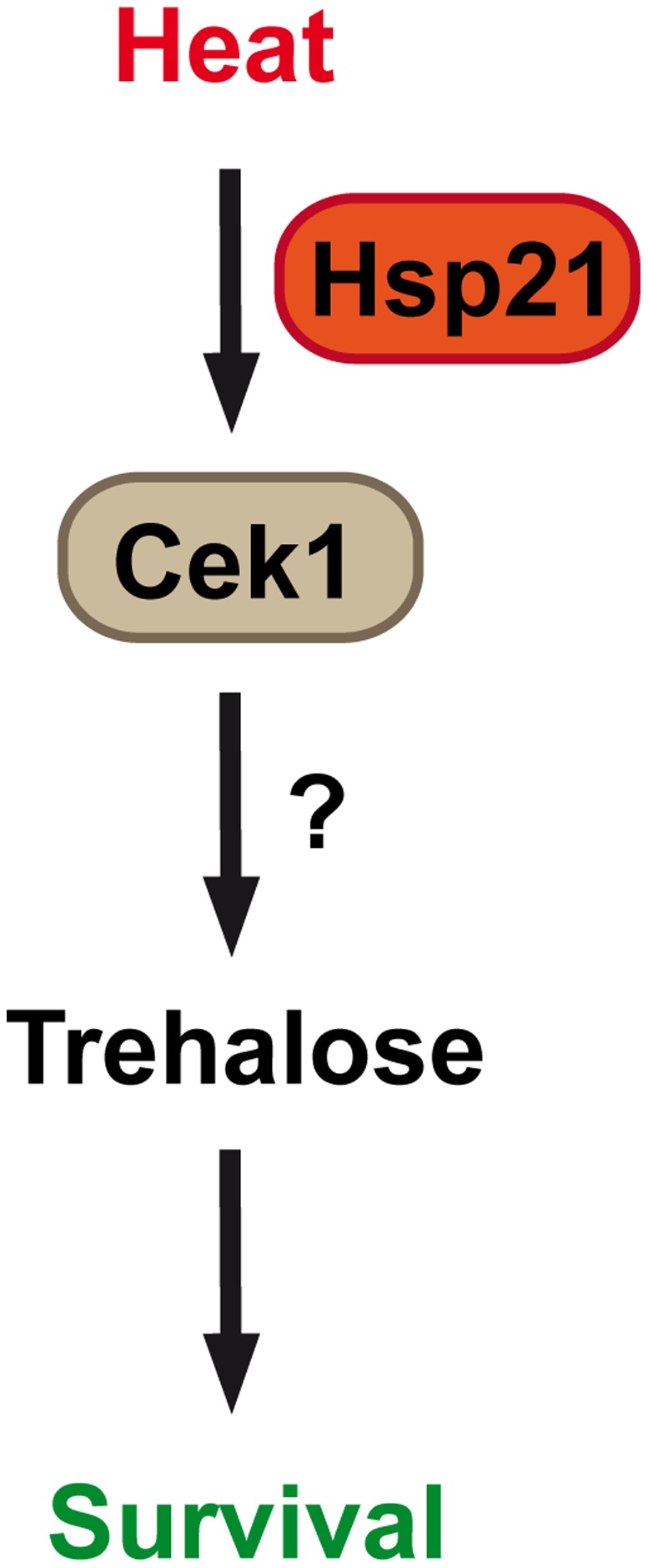
Model of Hsp21-dependent adaptation to elevated temperature. Heat stress induces Hsp21-dependent activation of Cek1, trehalose accumulation and thermal adaptation of *C. albicans*.

The dominant cellular function of Hsp21 appears to be thermal stress adaptation (Figure 9). The *hsp21*Δ/Δ mutant produced significantly less trehalose than the wild type under long-term elevated temperature. Trehalose is an important stress-protective molecule with chaperone-like functions and is specifically produced during heat and oxidative stress [85], [97], [98]. Therefore, Hsp21 is involved in thermal-induced trehalose synthesis, possibly via stabilizing metabolic enzymes such as Tps1–3. Interestingly, glycerol was over-produced by *hsp21*Δ/Δ cells in response to thermal stress. This directly demonstrates that, although incapable of growth, *hsp21*Δ/Δ cells were metabolically active under thermal stress and indicates that Hps21 rather fine-tunes the cellular balance of stress protectant molecules in response to environmental conditions.

In agreement with these conclusions, mutants defective in genes encoding key metabolic enzymes for the synthesis of trehalose (Tps1, Tps2) phenocopied *HSP21*-deletion. The trehalose 6-phosphate synthase Tps1 and the trehalose 6-phosphate phosphatase Tps2 form a complex together with the stabilizing proteins Tps3 and Tsl1 [99]. A *tps1*Δ/Δ mutant has previously been shown to be defective in trehalose production, hyphal formation, resistance to oxidative stress and virulence *in vivo* [89], [98], [100]. Interestingly, *tps1*Δ/Δ did not grow at 42°C on glucose but grew normally on glycerol [89]. Disruption of *TPS2* in *C. albicans* leads to defective trehalose accumulation, thermosensitivity, sensitivity to oxidative stress, and attenuated virulence in mice [88], [101], [102]. However, the capacity for hyphal formation was unaffected in this mutant [88]. Therefore, the thermal sensitivity of the *hsp21*Δ/Δ mutant is most likely due to impaired trehalose synthesis.

Interestingly, the defect of the *hsp21*Δ/Δ mutant to grow at 42°C could be completely bypassed by simultaneously applying osmotic stress. In a recent publication it has been shown for the filamentous fungus *Aspergillus fumigatus*, that deletion of the UPR-regulating transcription factor HacA results in a similar phenotype, i.e. inability of a Δ*hacA* mutant to grow at elevated temperatures (45°C) which is reversed by supplementation of the medium with sorbitol or KCl [65]. The authors conclude that osmotic stabilization of the medium compensates for reduced cell wall integrity of the Δ*hacA* mutant. However, *C. albicans hsp21*Δ/Δ did not exhibit defects in cell wall integrity (Figure 2A and data not shown) and mutant cells did not lyse upon thermal stress, but rather remained viable and metabolically active. Osmotic stabilization of cellular integrity, in this case appears improbable. An alternative explanation is that osmotic stress of *hsp21*Δ/Δ resulted or the induction of other heat shock protein(s) (such as *HSP12* [49]), or stress responsive pathways (such as Cek1, see below) thereby compensating for the lack of Hsp21.

*HSP21* has been shown to be upregulated in the absence of the adenylyl cyclase Cyr1 [103]. Therefore, *HSP21* lies downstream of the cyclic AMP pathway.

To determine which pathway(s) Hsp21 functions in, we performed a systematic Western blot analysis of the three main stress responsive MAP kinase pathways (Mkc1-, Cek1- and Hog1-mediated pathways) in *C. albicans* wild type and *hsp21*Δ/Δ strains under a range of stress conditions. Of the three MAP kinases, Cek1 phosphorylation in response to thermal stress was found to be Hsp21-dependent. Significantly, dual challenge of cells with osmotic and thermal stress bypassed Hsp21-dependent Cek1 phosphorylation. Therefore, the Cek1 phosphorylation state of *C. albicans* directly correlates with the ability to grow under elevated temperatures. In line with this, *C. albicans CEK1* has previously been shown to be induced by high temperatures [104]. These data suggest that Hsp21 functions upstream of Cek1 in a temperature-responsive pathway. It remains to be investigated whether Cek1 is responsible for activation of trehalose synthesis in response to elevated temperatures.

Furthermore, Cek1 has been shown to be required for hyphal formation on solid Spider, SLAD and serum agar, and for full virulence in a mouse model of systemic candidiasis [105]. These phenotypes correlate well with those observed for *hsp21*Δ/Δ. In contrast to deletion of *HSP21*, however, *cek1*Δ/Δ was found to be unattenuated in resisting killing by neutrophils and macrophages [92], [106], indicating that Hsp21 has further cellular functions, possibly by stabilizing additional client proteins.

Deletion of Hsp21 also affected hyphal growth and hypha-associated processes. The *hsp21*Δ/Δ mutant formed shorter hyphae, smaller hyphal colonies than the wild type and exhibited reduced capacity to invade semi-solid agar. These morphological defects are likely to have contributed at least partially to the reduced damage capacity of *hsp21*Δ/Δ during infection of endothelial and oral epithelial cell monolayers. Although the epithelial/endothelial adhesion and initial invasion properties of the mutant were unaffected, the reduced damage capacity of the *hsp21*Δ/Δ mutant may be due to a compromized capacity to undergo subsequent inter-cellular invasion [82].

Importantly, the *hsp21*Δ/Δ mutant strain was avirulent in a mouse model of hematogenously disseminated candidiasis and displayed attenuated virulence in an alternative embryonated egg infection model. Based on our detailed functional analysis of *HSP21*, a number of mechanisms are likely to account for the reduced virulence of *hsp21*Δ/Δ.

Mutants with morphological defects generally exhibit reduced virulence in both murine and *in ovo* infection models [84], [107]. Therefore, it is possible that reduced hyphal formation *in vivo* may at least partially account for the mutant’s virulence attenuation.

Several studies have demonstrated a correlation between reduced capacity to damage host cells and attenuated virulence [22], [108], [109], [110]. Infection of embryonated eggs was performed via the chorio-allantoic membrane (CAM). The CAM is a thin, highly vascularized membrane composed of two epithelial cell layers, held together by connective tissue [111]. Therefore, the reduced capacity of *hsp21*Δ/Δ to damage epithelial and endothelial cells is likely to have contributed to attenuated virulence *in ovo*. Similarly, following murine intravenous infection, *C. albicans* must traverse the endothelial lining of blood vessels in order to infect deeper organs. It is therefore possible that the reduced endothelial damage potential of *hsp21*Δ/Δ may account for decreased virulence during disseminated candidiasis.

Finally, the role of Hsp21 in adaptation to environmental stresses likely plays a crucial role in *C. albicans* virulence. *hsp21*Δ/Δ was unable to grow at elevated temperature, exhibited greatly increased sensitivity to oxidative stress and was killed more efficiently by human neutrophils. As neutrophils play a key role in killing *C. albicans* [52], it seems likely that the increased sensitivity of *hsp21*Δ/Δ to these phagocytes contributed to the strongly reduced virulence of the mutant. Moreover, host cells and tissues are known to induce stress-defensive mechanisms, such as the generation of reactive oxygen (ROS) and/or nitrogen species (RNS). Although a febrile response is unlikely to reach the high temperatures used for *in vitro* thermal stress experiments, it is likely that *in vivo*, a combination of stresses act simultaneously on invading microbes. Indeed, histopathologic examinations, together with CFU counts of end-point surviving kidney homogenates demonstrated that very few fungal cells remained in the kidneys of mice infected with *hsp21*Δ/Δ. We therefore conclude that, unlike wild type and *hsp21*Δ/Δ::*HSP21* strains, *hsp21*Δ/Δ survived poorly in the hostile milieu of the mammalian host.

In summary, this study represents the first characterization of a small heat shock protein (Hsp21) in the human fungal pathogen *C. albicans* and establishes its role in adaptation to distinct environmental stresses via cellular trehalose homeostasis and Cek1 activation, immune evasion and virulence.

## Materials and Methods

### Ethics Statement

All animal experiments were in compliance with the German animal protection law and were approved (permit no. 03–007/07) by the responsible Federal State authority (Thüringer Landesamt für Lebensmittelsicherheit und Verbraucherschutz) and ethics committee (beratende Komission nach § 15 Abs. 1 Tierschutzgesetz). The use of human primary cells in this study was conducted according to the principles expressed in the Declaration of Helsinki. All protocols used in this study were approved by the local ethics committee of the University of Jena under the permit no. 2207-01/08. Written informed consent was provided by all study participants.

### Strains and Growth Conditions

*C. albicans* strains used and constructed in the present study are listed in Table 2. The triple-auxotrophic strain BWP17 complemented with plasmid CIp30 [112] was used as wild type control in all experiments. Strains were routinely grown on YPD agar [1% yeast extract, 2% bacto-peptone, 2% D-glucose, 2% agar] or SD minimal medium agar [2% dextrose, 0.17% yeast nitrogen base, 0.5% ammonium sulfate, 2% agar]. Overnight liquid cultures were grown in YPD or SD medium in a shaking incubator at 30°C and 180 rpm. Transformants were selected on SD agar supplemented with 20 µg ml^-1^ arginine, histidine and/or uridine as required. *E. coli* was cultivated on LB agar [1% bacto-tryptone, 0.5% yeast extract, 1% NaCl, 2% agar]. Overnight cultures of *E. coli* were grown in a shaking incubator at 37°C and 210 rpm. For selection purposes 50 µg/ml Ampicillin were added to the solid or liquid LB medium. For growth curves overnight YPD cultures grown at 30°C were diluted to an OD_600_ of 0.2 in 200 µl final volume of the desired medium. Growth of the strains was then recorded by measuring the OD_600_ in a 30 min interval for up to 60 hours in a ELISA reader (Infinite M200, Tecan) [113]. Experiments were performed at least twice in duplicate. Similar results were obtained and representative curves are shown.

**Table 2:** *C. albicans* strains used in this study.

*Strain*	*Genotype*	*Reference*
SC5314	*Candida albicans* wild type	[125]
BWP17	*ura3 ::λimm434/ura3 ::λimm434 arg4::hisG/arg4::hisG his1::hisG/his1::hisG*	[115]
BWP17+ CIp30	*ura3 ::λimm434/ura3 ::λimm434 arg4::hisG/arg4::hisG his1::hisG/his1::hisG +* CIp30	[112]
*hsp21Δ*	*orf19.822Δ::ARG4/ORF19.822*	This study
*hsp21Δ/Δura*	*orf19.822Δ::ARG4/orf19.822Δ::HIS1*	This study
*hsp21Δ/Δ*	*orf19.822Δ::ARG4/orf19.822Δ::HIS1+* CIp10 *(URA3)*	This study
*hsp21Δ/Δ::HSP21*	*orf19.822Δ::ARG4/orf19.822Δ::HIS1+* CIp10 *(ORF19.822, URA3)*	This study

### Strain Construction

The *hsp21*Δ/Δ homozygous null mutant was generated using a PCR-based gene disruption technique [114]. Starting with the Arg-, His- and Ura-auxotrophic parental strain BWP17 [115], the complete open reading frames (ORFs) of both *HSP21* alleles were replaced with polymerase chain reaction (PCR)-amplified *ARG4* and *HIS1* disruption cassettes flanked by 104 base pairs of target homology region. Two sequential transformations using the improved lithium-acetate method [116] were applied for both disruption cassettes. Primers HSP21-FG and HSP21-RG (Table S2) were used for generation of the *ARG4* and *HIS1* deletion cassettes with the pFA-*ARG4* and pFA-*HIS1* plasmids as templates [114]. Resultant deletion cassettes were used to sequentially delete both copies of *HSP21* (orf19.822). The resultant Ura-auxotrophic mutant was rendered prototrophic for uridine by transformation with the NcoI-linearized plasmid CIp10, which harbors the *URA3* gene and stably integrates at the *RPS10* locus [117]. The correct deletion of both alleles and integration of CIp10 was verified by colony PCR using target gene and disruption/integration cassette flanking and internal primers: HSP21-F1, HSP21-R1, ARG4-F1, ARG4-R1, HIS1-F1, HIS1-R1, URA3-F2 and RPF-F1 (Table S2), respectively.

Additionally, Southern blot analysis (Figure S2) using a 269 base-pair PCR product, generated with the primers HSP21-F2 and HSP21-R2 (Table S2) from *C. albicans* SC5314 genomic DNA, as a probe on *Hind*II-digested genomic DNA was used to confirm deletion of *HSP21*/orf19.822.

For the generation of a *hsp21*Δ/Δ::*HSP21-*reconstituted strain, the open reading frame of *HSP21* as well as 406 base pairs of upstream and 190 base pairs of downstream sequence were amplified from SC5314 genomic DNA with the Phusion High-Fidelity DNA Polymerase Kit (Finnzymes) using the *Hind*III restriction site containing primers HSP21rec-F1 and HSP21rec-R1 (Table S2). The resulting PCR product was first digested with *Hind*III and then further purified with the QIAquick PCR Purification Kit (Qiagen). In parallel 0.3 µg µl^-1^ of plasmid CIp10 was digested with *Hind*III and the restriction enzyme then heat inactivated by an incubation at 65°C for 20 min. The linearized plasmid was dephosphorylated with calf intestinal alkaline phosphatase (New England BioLabs) and gel extracted using the QIAquick Gel Extraction Kit (Qiagen). The *HSP21* insert and CIp10 vector were then ligated for 30 min at 22°C using the Rapid DNA Ligation Kit (Fermentas). Five µl of ligation product was used for the transformation of *E. coli* DH5α and positive clones were selected on LB agar plates supplemented with 50 µg ml^-1^ Ampicillin. Plasmid CIp10 carrying *HSP21* was re-isolated using plasmid miniprep (peqlab) and midiprep (Qiagen) kits and confirmed by control digestions with *Hind*III, *Sac*I and *Spe*I and sequencing. The final plasmid was then digested with *Nco*I prior to transformation into the uridine auxotrophic *C. albicans* strain *hsp21*Δ/Δ*ura^-^* (Table 2). Positive clones were selected on SD agar plates without amino acids. Correct integration was verified by PCR on whole yeast colonies using primers RPF-F1 and URA3-F2 (Table S2).

### Susceptibility to Stressors

Aliquots of overnight YPD cultures were washed twice in phosphate buffered saline (PBS) and 10-fold serial dilutions in 5 µl (covering a range of 10^6^–10^1^ cells) were spotted onto YPD agar containing 30 mM DTT (Roth) or SD agar containing 0.4 mM menadione (Sigma), 1.5 M NaCl or 450 µg ml^-1^ Congo red (Sigma) and incubated at 37°C for 3–4 days. Plates incubated at 42°C were photographed after 4–6 days. Heat shock was performed by incubating serial 10-fold dilutions (range 10^6^–10^1^) at 50°C for 15 min, followed by an incubation on YPD agar for 2 days at 37°C. Each experiment was repeated at least twice. Representative pictures are shown.

### Growth under Nutrient Limitation

For growth assays under nutrient limitation, agar containing 0.67% yeast nitrogen base plus ammonium sulphate without amino acids (Difco) was supplemented with 2% glucose, potassium acetate or citrate as sole carbon source. Solid yeast nitrogen base agar without ammonium sulphate and amino acids (Difco) was supplemented with 100 µg ml^-1^ proline or pantothenate as sole carbon and nitrogen source. Spot dilution assays (range 10^6^–10^1^) were prepared and plates were incubated at 37°C for 3–7 days depending on the carbon and nitrogen source.

### Measurement of Intracellular Glycerol, Glycogen and Trehalose Levels

Determination of intracellular glycerol content was performed with the EnzyChrom Glycerol Assay Kit (Bio Assay Systems) as previously described [118]. Briefly, cells were grown to stationary phase at 30°C or 42°C, washed twice with water, and resuspended in water. Cells were then lysed by incubating at 95°C for 10 min and the supernatant was used for colorimetric analysis. Intracellular glycerol levels were normalized to the wet weight of each cell pellet. The experiment was performed twice in duplicate and a third time as a single reaction.

Estimation of intracellular glycogen levels was performed using the iodine vapour technique [86], [87]. Briefly, 20 µl of stationary phase cells grown in YPD were spotted on appropriate agar plates, and incubated for 24 hours at 37°C or 42°C. Colonies were then exposed to iodine vapour for 5 minutes and immediately photographed. The experiment was repeated twice in duplicate.

Measurement of intracellular trehalose levels was performed based on previous studies [86], [119]. Briefly, cells were grown to stationary phase at 30°C or 42°C, washed twice with chilled water, and resuspended in water. Lysis of the cells was achieved by incubating at 95°C for 30 minutes and the supernatant was used for enzymatic analysis. Trehalose was converted to glucose by addition of 0.15 U trehalase (Sigma) to the reactions (50 µl of sample, 100 µl 270 mM citric acid buffer ph 5.7) and incubating at 37°C for five hours. Glucose concentrations were then determined using the hexokinase glucose kit (Sigma) and adjusted based on reactions without trehalase. Total protein content was determined using the BCA protein assay (Pierce) and relative trehalose levels were based on nmol trehalose per mg cell protein. At least two biological replicates were performed per strain and condition.

### Hyphal Elongation

Hyphal elongation was investigated on solid water agar supplemented with 10% fetal bovine serum, on solid synthetic low-ammonium-dextrose (SLAD) medium, on solid Spider medium [76] or by embedding in YPS (2% saccharose) agar. Serum agar plates were incubated for 2, SLAD agar plates for 4 and Spider agar plates for 10 days at 37°C. Embedded plates were incubated at 25°C for 5 days. Experiments were performed twice in duplicate. Representative pictures are shown.

Hyphal lengths were measured based on previous studies [120]. Briefly, cells were grown overnight to stationary phase in SD medium, washed twice with water, and resuspended in water. Cell numbers were adjusted to 10^4^ cells per well in a 24-well cell culture plate in RPMI1640 or water supplemented with 10% serum, and incubated at 37°C for 4 hours in presence of 5% CO_2_. Hyphal lengths were then determined using an Inverse microscope (Leica). Experiments were performed in duplicate and repeated twice. The lengths of at least 50 hyphae was determined per replicon, strain and condition. Representative pictures are shown.

Induction of hyphal elongation using host cells was performed by preparation of a monolayer of oral epthelial cells (TR146) and infecting it with 10^5^ *C. albicans* cells. Monolayers were incubated at 37°C for 3 hours in a 5% CO_2_ atmosphere and hyphal cells were then differentially stained according to the invasion assay described below. Hyphal lengths were determined by fluorescence microscopy (Leica DM5500B, Leica DFC360 FX) with the Leica Application Suite (LAS) software. At least 100 *C. albicans* cells were examined for each strain and all experiments were performed in duplicate at least twice. Representative pictures are shown.

### Western Blot Analysis

Western blot analysis for detection of phosphorylated Cek1 and Mkc1 was performed as previously described [121], with some modifications. Briefly, overnight YPD cultures of the BWP17+CIp30 wild type, *hsp21*Δ/Δ and *hsp21*Δ/Δ::*HSP21* mutant strains were adjusted to OD 0.5 in 10 ml final volume and grown under the following conditions for 4 hours with shaking (180 rpm): (i) SD minimal medium at 30°C and 42°C, (ii) SD minimal medium with 450 µg/ml Congo red at 30°C, (iii) SD minimal medium with 0.5 M NaCl at 30°C, (iv) SD minimal medium with 50 mM menadione at 30°C, and (v) SD minimal medium with 0.5 M NaCl at 42°C. Cells were collected by centrifugation at 4°C and washed twice with cold lysis buffer containing 1×PBS, 3 mM KCl, 2.5 mM MgCl_2_, 0.1% Triton X-100, 50 mM NaF, 2 mM Na_3_VO_4_. Cell pellets were resuspended in cold lysis buffer (see above) containing a protease inhibitor cocktail (Roche). Cells were then mechanically disrupted by adding acid-washed glass beads and bead beating in a Precellys 24 homogenizer (peqlab). Protein concentrations were determined by BCA Protein Assay (Pierce). Protein samples (80 µg) were mixed with one-fourth volume of 4x sample buffer containing 125 mM Tris-HCl (pH 6.8), 50% glycerol, 4% SDS, 2.5% β-mercaptoethanol, and 0.02% bromophenol blue for SDS-PAGE. Samples were heated at 95°C for 5 min and then separated by SDS-PAGE using 12% acrylamide gels. Proteins were electro-transferred to Protran B85 nitrocellulose membranes (Whatman) and blocked with 5% BSA (Serva) in PBS with 0.05% tween. Blots were then probed with primary anti-phospho-p44/42 MAP kinase antibody (1∶1000, Cell Signaling Technology) and secondary goat anti-rabbit-horseradish peroxidase (HRP)-conjugated antibody (1∶2500, Santa Cruz), and developed using the Enhanced Chemiluminescent (ECL) SuperSignal West Dura kit (Thermo Scientific) according to the manufacturers’ instructions. Membranes were then stripped for 30 min at 50°C using a buffer containing 2% SDS, 125 mM Tris-HCl (pH 6.8) and 0.7% β-mercaptoethanol. Stripped membranes were then blocked with 5% BSA (Serva) in PBS with 0.05% tween and re-probed for α-tubulin (loading control) by using a primary rat anti-α-tubulin antibody (1∶1000, AbD Serotec) and a secondary goat anti-rat HRP-conjugated antibody (1∶2000, Santa Cruz), and developed as described above. Experiments were performed twice.

Western blot analysis for detection of phosphorylated Hog1 levels was performed as described above, but here, blots were probed with primary rabbit anti-Phospho-p38 MAP Kinase (Thr180/Tyr182) antibody (1∶1000 Cell Signaling Technology) and secondary anti-rabbit IgG horseradish peroxidase (HRP)-conjugated antibody (1∶2000, Cell Signaling Technology). Stripped membranes were re-probed for total Hog1 (loading control) by using a primary rabbit anti-Hog1 (y-215) antibody (1∶1000, Santa Cruz Biotechnology) and a secondary anti-rabbit IgG horseradish peroxidase (HRP)-conjugated antibody (1∶2000, Cell Signaling Technology), and developed as described above. Experiments were performed twice.

### Endothelial and Oral Epithelial Cells

The human buccal carcinoma derived epithelial cell line TR-146 (Cancer Research Technology, London) [122] and the human umbilical vein derived endothelial cell line HUVEC (ATCC CRL-1730, LGC Standards, Promocell) were routinely cultured and passaged in Dulbecco Modified Eagles Medium (DMEM) with 2 mM L-glutamine (PAA) supplemented with 10% heat inactivated (56°C for 10 min) fetal bovine serum (FBS, PAA). For experiments, TR146 cells were used during passage 10–20 and HUVEC cells during passage 10–40. Both cell lines were cultured in a humidified incubator at 37°C with 5% CO_2_ atmosphere. Cultivation medium was replaced by fresh medium every second day and accutase (PAA) was used for detaching cells after confluency had reached about 80–100%.

### Adherence Assay

Adherence assays were performed using ibidi µ-Slides VI ^0.4^ with six channels per slide. For adherence studies of *C. albicans* strains to human host cells 1.8×10^4^ endothelial or epithelial cells, respectively, were seeded per µ-slide channel and incubated for 3 days at 37°C and 5% CO_2_. Confluent monolayers were then infected with 1.5×10^4^ *C. albicans* cells per channel for 45 min. Monolayers were then thoroughly washed with PBS to remove un-adhered fungal cells and fixed with 4% paraformaldehyde. *C. albicans* cells were subsequently stained with calcofluor white and quantified by fluorescence microscopy (Leica DM5500B, Leica DFC360 FX). The number of adhered cells was determined by counting at least 50 high power fields of 200 µm×200 µm size. Experiments were performed in triplicate on three separate occasions.

### Invasion Assay

Invasion rates of the different *C. albicans* strains were determined as previously described [81]. Briefly, epithelial TR146 cells were grown to confluency on 15 mm diameter glass coverslips for 2–3 days. Monolayers were washed with PBS prior to infection. Infection was then performed by adding 10^5^ *C. albicans* yeast cells to the monolayers and incubating the plates for 3 hours at 37°C and 5% CO_2_. Next, epithelial cells were washed twice with PBS and fixed with 4% paraformaldehyde (Roth). Fungal cells were then stained for 45 min with fluorescein-conjugated concanavalin A (Con A) (Invitrogen). After washing with PBS, epithelial cells were permeabilized in 1% Triton X-100 in PBS for 15 min. Next, fungal cells were stained with calcofluor white. The coverslips were then rinsed three times with water and mounted with the cells upside down on microscope slides with ProLong Gold Antifade Reagent. Fluorescence microscopy was performed (Leica DM5500B, Leica DFC360 FX) using appropriate filter sets for detection of fluorescein-conjugated Con A (stains only the extracellular, non-invaded fungal elements) and calcofluor white (stains both invaded and non-invaded fungal elements). At least 100 *C. albicans* cells were examined for each strain and the invasion rate was expressed as percentage of invaded cells divided by the number of invaded plus non-invaded cells. Representative pictures were taken for each strain. All experiments were performed on three separate occasions.

### Damage Assay

Damage assays were performed by measuring the activity of lactate dehydrogenase (LDH) released from the cytosol of damaged cells into the surrounding supernatant using the Cytotoxicity Detection Kit (Roche Applied Science). TR146 or HUVEC cells were adjusted to 10^5^ cells ml^-1^ in DMEM with 10% FBS and 200 µl were seeded per well in 96 well plates (TPP). Plates were incubated at 37°C and 5% CO_2_ for 2 days until confluency had been reached. Cells were then washed twice with PBS and 100 µl DMEM with 2% FBS were added per well. For the *C. albicans* strains, aliquots of overnight YPD cultures were washed twice in PBS and 100 µl of 5×10^5^ cells ml^-1^ DMEM without FBS were added to the host cells. Controls included a medium only control, a low control with uninfected host cells and medium only and a high control with infected host cells and medium supplemented with 1% Triton X-100. Incubation was carried out at 37°C and 5% CO_2_ for 15 or 24 h. Measurement of LDH activity with the Cytotoxicity Detection Kit was performed according to the manufacturer’s manual. Absorbance of the samples was measured at 490 nm. Medium only and low control values were subtracted from all sample values. Damage was expressed as percentage of the high control, which was set to 100%. Each experiment was performed at least three times in triplicate.

### Neutrophil Assay

Neutrophils were isolated from blood of healthy human donors by a density gradient centrifugation using Histopaque 1077 and 1119 (Sigma, MO, USA) following the manufacturer's instructions. Polymorphonuclear cells (PMNs) were obtained after a centrifugation step at 700 g for 30 min at room temperature and then transferred to PBS. The remaining erythrocytes were lysed in a lysis buffer (0,83% NH_4_Cl, 10 mM HEPES, pH 7.0), the PMNs were washed once in PBS and resuspended in 1 ml RPMI1640+5% FBS. For investigating killing of *C. albicans* by neutrophils, 100 µl of fungal overnight cultures were collected and washed twice with PBS. *C. albicans* cells were then opsonized with 50% human serum for 30 min at 37°C. Following centrifugation and resuspension in PBS, 10^5^ cells ml^-1^ were inoculated into RPMI1640+5% FBS. Neutrophils and fungal cells were then mixed in a ratio of 10∶1 (final volume: 400 µl) and incubated for 3 hours at 37°C in presence of 5% CO_2_. Neutrophils were lysed by addition of 100 µl 0.25% SDS at 30°C in order to release phagocytosed *C. albicans* cells. After addition of 900 µl pre-chilled water and 20 U of DNase-1, cells were incubated for 15 min at 30°C. Following preparation of appropriate dilutions, aliquots were spread in duplicate on YPD and incubated for 24 hours at 37°C. At least three independent experiments were performed.

### Embryonated Chicken Egg Infection Model

Egg infection experiments were performed as described previously [83], [123]. Briefly, fertilized chicken eggs were retrieved from a local producer and stored at 8°C not longer than 7 days before starting with the incubation. The eggs were incubated in a specialized incubator (BSS 300, Grumbach, Germany) at 37.6°C and 50–60% relative humidity before infection and turned four times a day starting on the fourth day of incubation. After confirming vitality of the embryos by candling, eggs were infected on top of the chorio-allantoic membrane (CAM) following 10 days of initial incubation, as previously described [83]. Survival of eggs was then monitored for up to seven days by candling the eggs at least twice a day. 20 eggs were infected per *C. albicans* strain and each experiment was performed at least twice. Survival data were visualized as Kaplan-Meyer curves.

### Mouse Model of Hematogenously Disseminated Candidiasis

Five to six weeks old female Balb/C mice (*Mus musculus*) (18–20 g; Charles River, Germany) were used for the experiments. The animals were housed in groups of five in individually ventilated cages and cared for in accordance with the principles outlined in the *European Convention for the Protection of Vertebrate Animals Used for Experimental and Other Scientific Purposes* (http://conventions.coe.int/Treaty/en/Treaties/Html/123.htm). Mice were challenged intravenously on day 0 with 5×10^5^ cfu in 200 µl PBS via the lateral tail vein. The health status of the mice was examined at least twice a day by a veterinarian. Body surface temperature and body weight were recorded once a day. Mice showing severe signs of illness like isolation from the group, apathy, hypothermia and drastic weight loss, were anaesthetized by application of 200 µl ketamine hydrochloride (50 mg ml^-1^) prior to blood collection by heart puncture. Gross pathological alterations were recorded during necropsy. For histology, left kidneys were collected and fixed with buffered formalin and paraffin-embedded sections were stained with Periodic acid-Schiff (PAS) according to standard protocols.

### Statistics

Differences in damage of endothelial and oral epithelial cells by the different *C. albicans* strains were compared by two-tailed, type three Student’s t-test. The statistical analysis for the susceptibility of *C. albicans* strains to killing by neutrophils was performed using Turkey’s Multiple Comparison test. Differences in survival of eggs or mice infected with the different *C. albicans* strains were evaluated by Log-rank (Mantel-Cox) and Gehan-Breslow-Wilcoxon tests. *P*-values ≤0.05 were considered to be statistically significant. All statistical tests were performed using GraphPad Prism version 5.00.

## Supporting Information

Figure S1 ***In silico*** **analysis of orf19.822.** (A) Phylogram for *C. albicans* orf19.822. The phylogram was generated according to [124]. Percentages represent identity of the respective orthologues to *C. albicans* orf19.822. (B) orf19.822 promoter region. Predicted heat shock elements and non-standard HSE are shown in bold underlined. Within these, the characteristic repetitive GAA and TTC triplets are shown in green, the variable base pairs are depicted in black. The stress-responsive element is represented in bold black characters. The putative TATA-Box is marked with black characters and is surrounded by a box.
(TIF)

Figure S2 **Deletion of both** ***HSP21*** **alleles.** The correct deletion of *HSP21* was confirmed by Southern blot analysis. Strains BWP17 (Wt), *hsp21*Δ, *hsp21*Δ/Δ*ura^-^* and *hsp21*Δ/Δ were analyzed. A 269 base-pair (bp) PCR product, with *C. albicans* SC5314 genomic DNA as template, was used as a probe on *Hind*II-digested genomic DNA. (A) Expected band sizes are: 727 bp (wild type *HSP21*), 1617 bp (*ARG4*-deletion-cassette) and 2098 bp (*HIS1*-deletion-cassette). (B) Southern blot.
(TIF)

Figure S3 **Complementation of the** ***hsp21*** **Δ/Δ mutant with** ***HSP21*** **restores** ***C. albicans*** **capacity to damage oral epithelial cells** ***in vitro*** **.** Monolayers of human-derived oral epithelial cells were infected with *C. albicans* wild type (Wt), *hsp21*Δ/Δ mutant and *hsp21*Δ/Δ::*HSP21* complemented mutant cells for 15 hours. Host cell damage was then determined by measuring lactate dehydrogenase (LDH) levels. Results are the mean ± SD of at least three independent experiments, each performed in triplicate. *P<0.0001 compared with the wild type and *hsp21*Δ/Δ::*HSP21* complemented strain.
(TIF)

Figure S4 ***hsp21*** **Δ/Δ has normal adherence and invasion properties upon contact with host cells.** (A) A *hsp21*Δ/Δ mutant has similar adherence properties to human-derived endothelial cells as the wild type. Adherence assays were performed using ibidi µ-Slides VI ^0^.^4^. Confluent endothelial cell monolayers were infected with 1.5×10^4^ *C. albicans* cells for 45 min. Monolayers were then thoroughly washed with PBS to remove unattached fungal cells and fixed with 4% paraformaldehyde. *C. albicans* cells were subsequently stained with calcofluor white and quantified by fluorescence microscopy. The number of adhered cells was determined by counting at least 50 high power fields of 200×200 µm. Results are the mean ± SEM of three independent experiments, each performed in triplicate. (B) Invasion of *hsp21*Δ/Δ mutant cells into human-derived epithelial cells is comparable to that of the wild type. Monolayers of confluent epithelial cells were infected with 10^5^ *C. albicans* yeast cells and incubated for 3 hours at 37°C and 5% CO_2_. After washing with PBS, cells were fixed with 4% paraformaldehyde. Fungal cells were stained for 45 min with fluorescein-conjugated concanavalin A. Epithelial cells were then permeabilized with 1% Triton X-100. Next, fungal cells were stained with calcofluor white. Fluorescence microscopy was performed using appropriate filter sets for detection of fluorescein-conjugated Con A (stains only the extracellular, non-invaded fungal elements) and calcofluor white (stains invaded and non-invaded fungal elements). At least 100 *C. albicans* cells were examined for each strain and the percentage invasion calculated. Results are the mean ± SEM of three independent experiments, with two of them performed in duplicate and one as a single quantification.
(TIF)

Figure S5 **The** ***hsp21*** **Δ/Δ mutant has attenuated virulence in an embryonated egg infection model.** 10-day old embryonated hen eggs were infected with either the wild type (Wt), the *hsp21*Δ/Δ mutant or the *hsp21*Δ/Δ::*HSP21* complemented strain (n = 20 eggs per *C. albicans* strain). Survival of the eggs was then monitored daily by candling for a total of 7 days. Results are the mean of at least two independent experiments per strain. *P<0.0001 compared with eggs either infected with the wild type or *hsp21*Δ/Δ::*HSP21* complemented strain.
(TIF)

Table S1 Transcriptional regulation of *C. albicans* orf19.822 (*HSP21*).
(DOC)

Table S2 Primers used in this study.
(DOC)
