# Supplementary figures and images for: Small but Crucial: The Novel Small Heat Shock Protein Hsp21 Mediates Stress Adaptation and Virulence in Candida albicans
Source: PLoS One. 2012 Jun 7;7(6):e38584. doi: 10.1371/journal.pone.0038584 (PMC3369842; doi:10.1371/journal.pone.0038584)

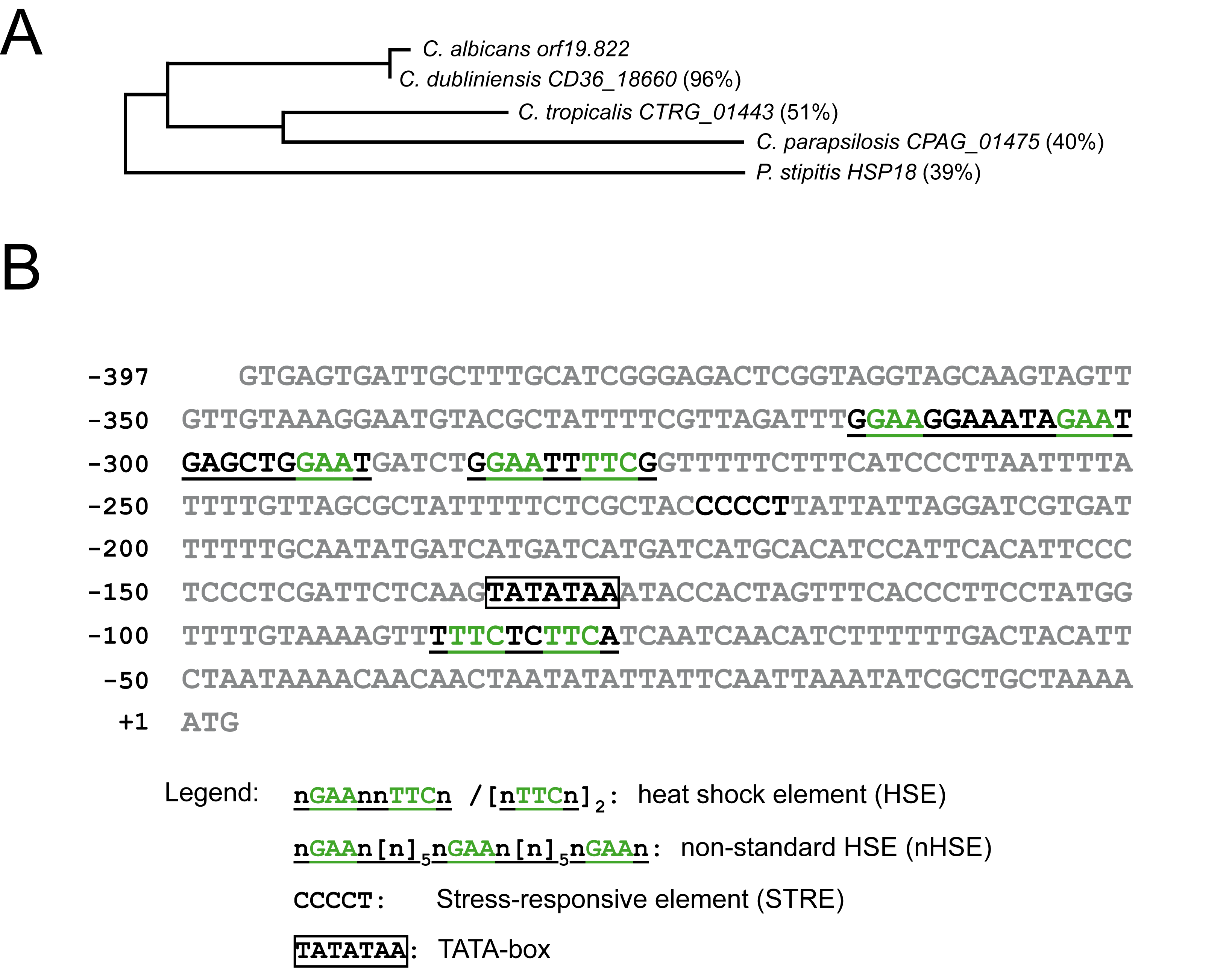

Supplement: Figure S1 — In silico analysis of orf19.822. (A) Phylogram for C. albicans orf19.822. The phylogram was generated according to [124]. Percentages represent identity of the respective orthologues to C. albicans orf19.822. (B) orf19.822 promoter region. Predicted heat shock elements and non-standard HSE are shown in bold underlined. Within these, the characteristic repetitive GAA and TTC triplets are shown in green, the variable base pairs are depicted in black. The stress-responsive element is represented in bold black characters. The putative TATA-Box is marked with black characters and is surrounded by a box. (TIF) [file pone.0038584.s001.tif]

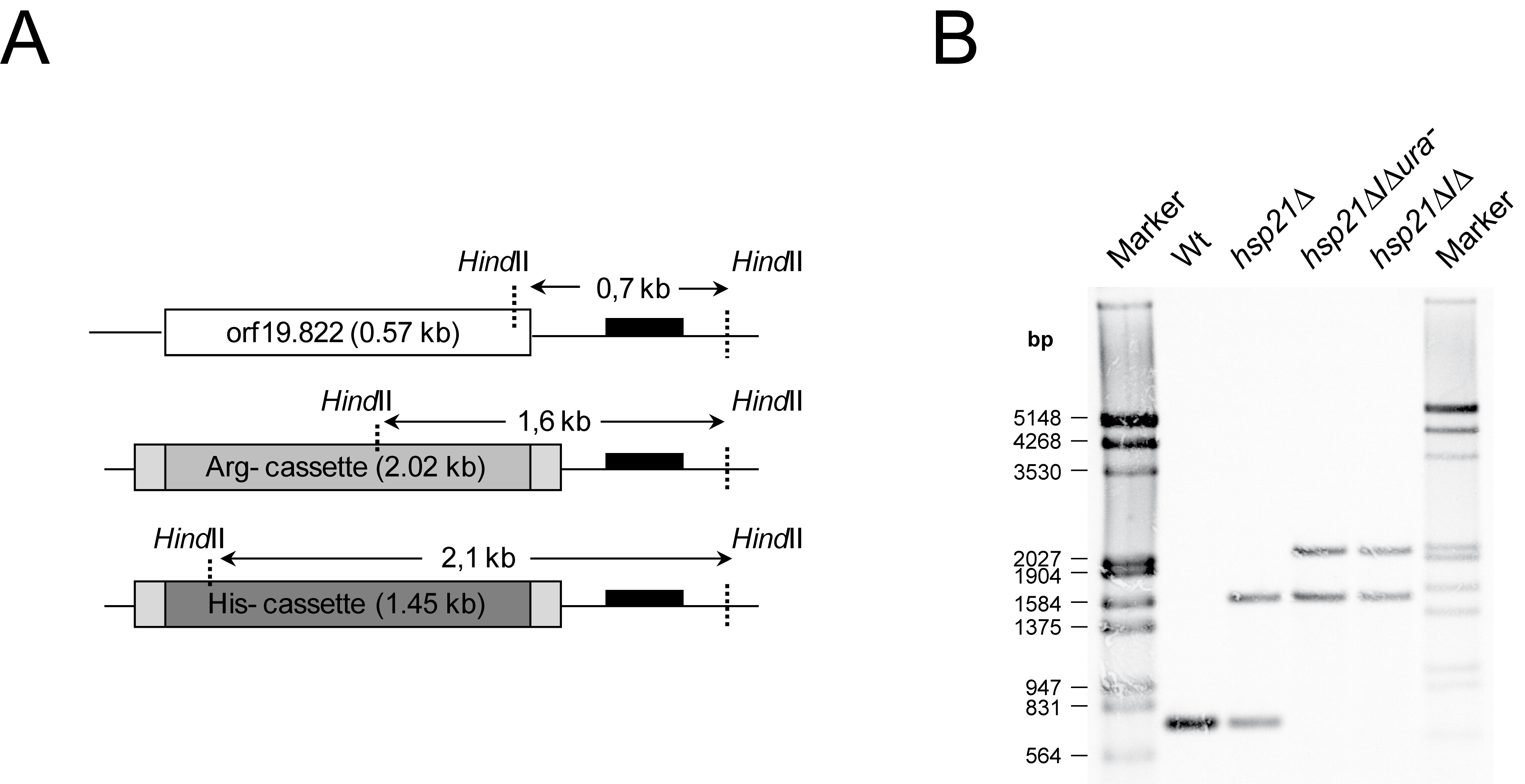

Supplement: Figure S2 — Deletion of both HSP21 alleles. The correct deletion of HSP21 was confirmed by Southern blot analysis. Strains BWP17 (Wt), hsp21Δ, hsp21Δ/Δura- and hsp21Δ/Δ were analyzed. A 269 base-pair (bp) PCR product, with C. albicans SC5314 genomic DNA as template, was used as a probe on HindII-digested genomic DNA. (A) Expected band sizes are: 727 bp (wild type HSP21), 1617 bp (ARG4-deletion-cassette) and 2098 bp (HIS1-deletion-cassette). (B) Southern blot. (TIF) [file pone.0038584.s002.tif]

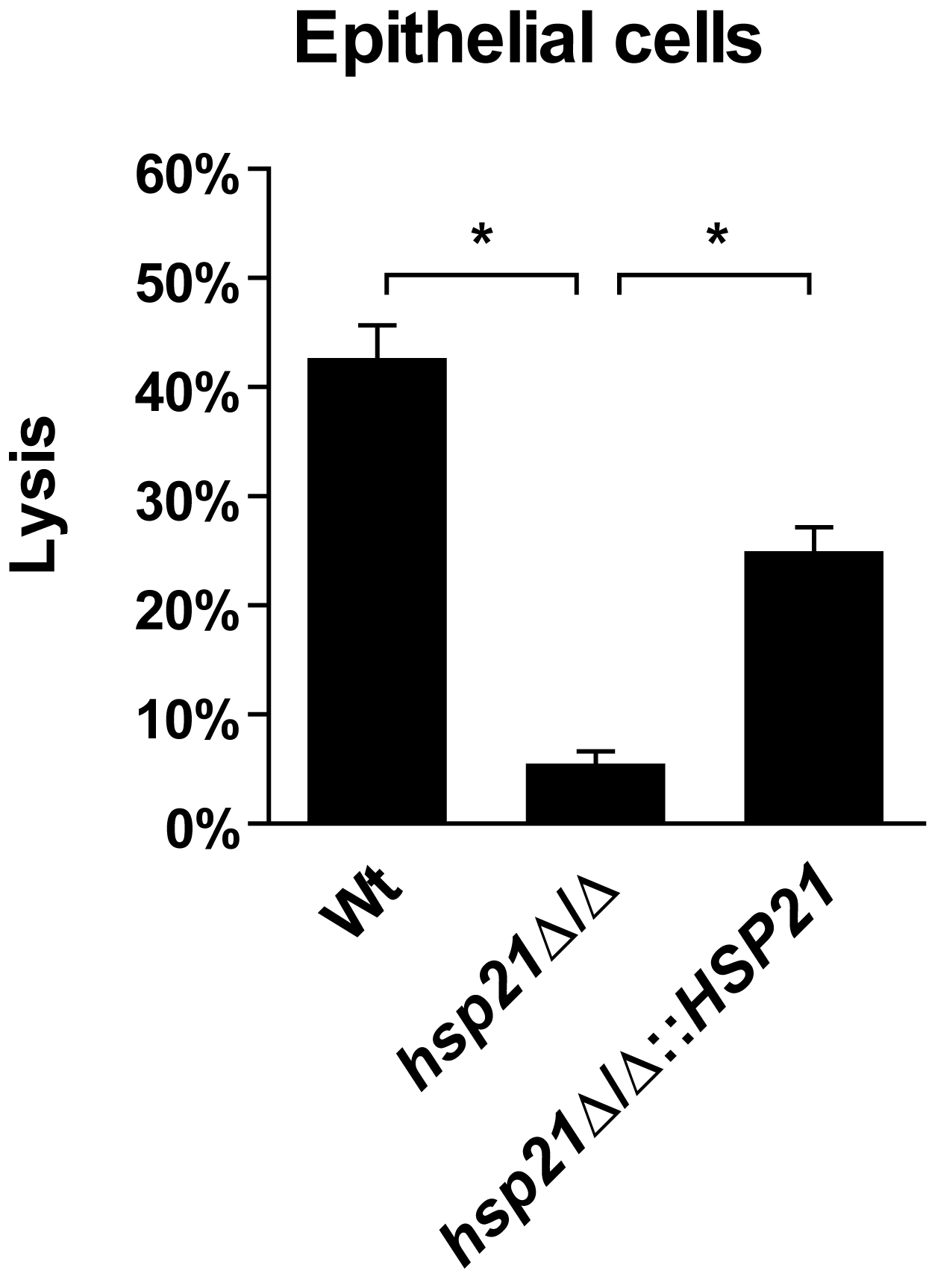

Supplement: Figure S3 — Complementation of the hsp21 Δ/Δ mutant with HSP21 restores C. albicans capacity to damage oral epithelial cells in vitro . Monolayers of human-derived oral epithelial cells were infected with C. albicans wild type (Wt), hsp21Δ/Δ mutant and hsp21Δ/Δ::HSP21 complemented mutant cells for 15 hours. Host cell damage was then determined by measuring lactate dehydrogenase (LDH) levels. Results are the mean ± SD of at least three independent experiments, each performed in triplicate. *P<0.0001 compared with the wild type and hsp21Δ/Δ::HSP21 complemented strain. (TIF) [file pone.0038584.s003.tif]

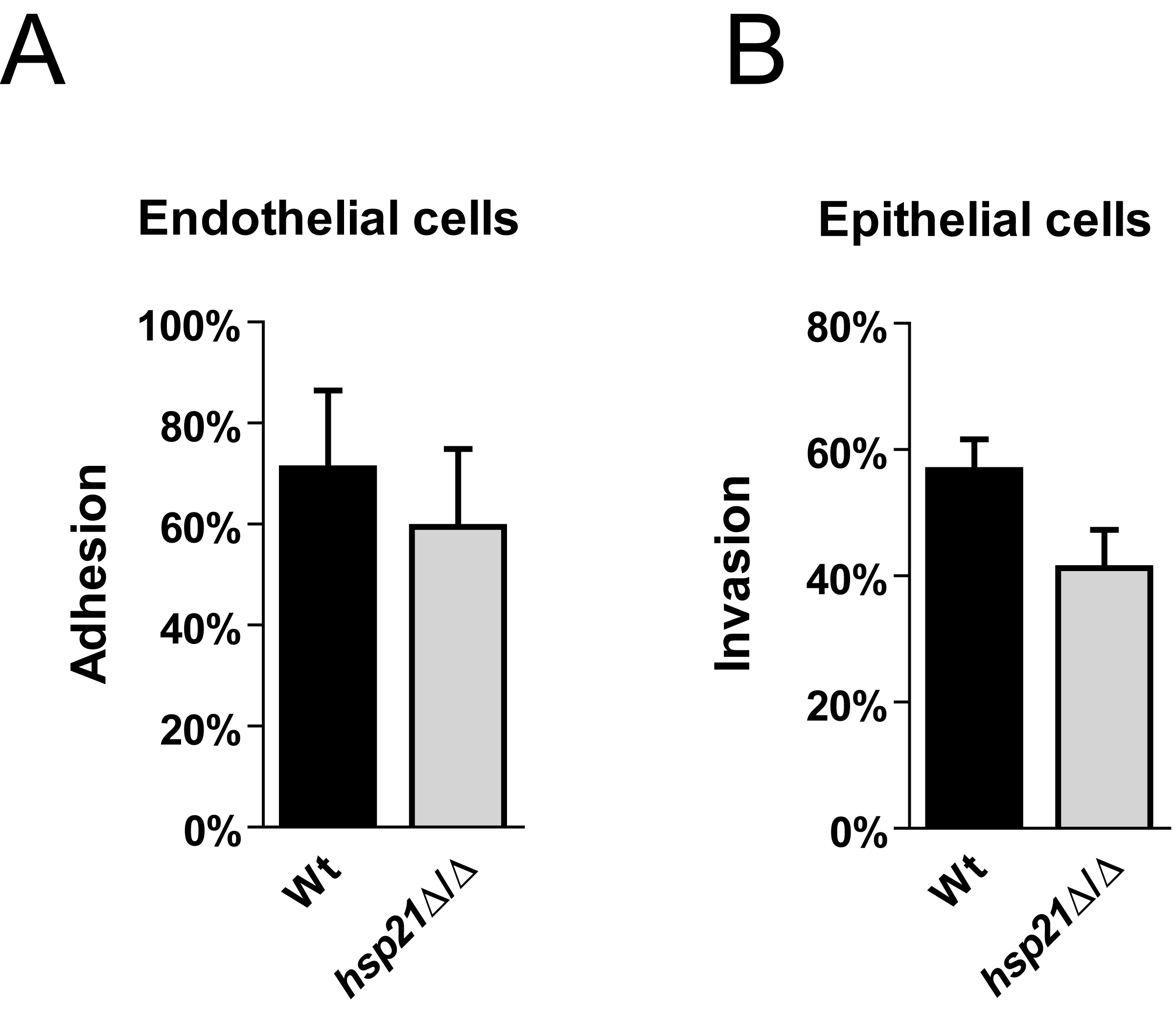

Supplement: Figure S4 — hsp21 Δ/Δ has normal adherence and invasion properties upon contact with host cells. (A) A hsp21Δ/Δ mutant has similar adherence properties to human-derived endothelial cells as the wild type. Adherence assays were performed using ibidi µ-Slides VI 0.4. Confluent endothelial cell monolayers were infected with 1.5×10^4^ C. albicans cells for 45 min. Monolayers were then thoroughly washed with PBS to remove unattached fungal cells and fixed with 4% paraformaldehyde. C. albicans cells were subsequently stained with calcofluor white and quantified by fluorescence microscopy. The number of adhered cells was determined by counting at least 50 high power fields of 200×200 µm. Results are the mean ± SEM of three independent experiments, each performed in triplicate. (B) Invasion of hsp21Δ/Δ mutant cells into human-derived epithelial cells is comparable to that of the wild type. Monolayers of confluent epithelial cells were infected with 105 C. albicans yeast cells and incubated for 3 hours at 37°C and 5% CO2. After washing with PBS, cells were fixed with 4% paraformaldehyde. Fungal cells were stained for 45 min with fluorescein-conjugated concanavalin A. Epithelial cells were then permeabilized with 1% Triton X-100. Next, fungal cells were stained with calcofluor white. Fluorescence microscopy was performed using appropriate filter sets for detection of fluorescein-conjugated Con A (stains only the extracellular, non-invaded fungal elements) and calcofluor white (stains invaded and non-invaded fungal elements). At least 100 C. albicans cells were examined for each strain and the percentage invasion calculated. Results are the mean ± SEM of three independent experiments, with two of them performed in duplicate and one as a single quantification. (TIF) [file pone.0038584.s004.tif]

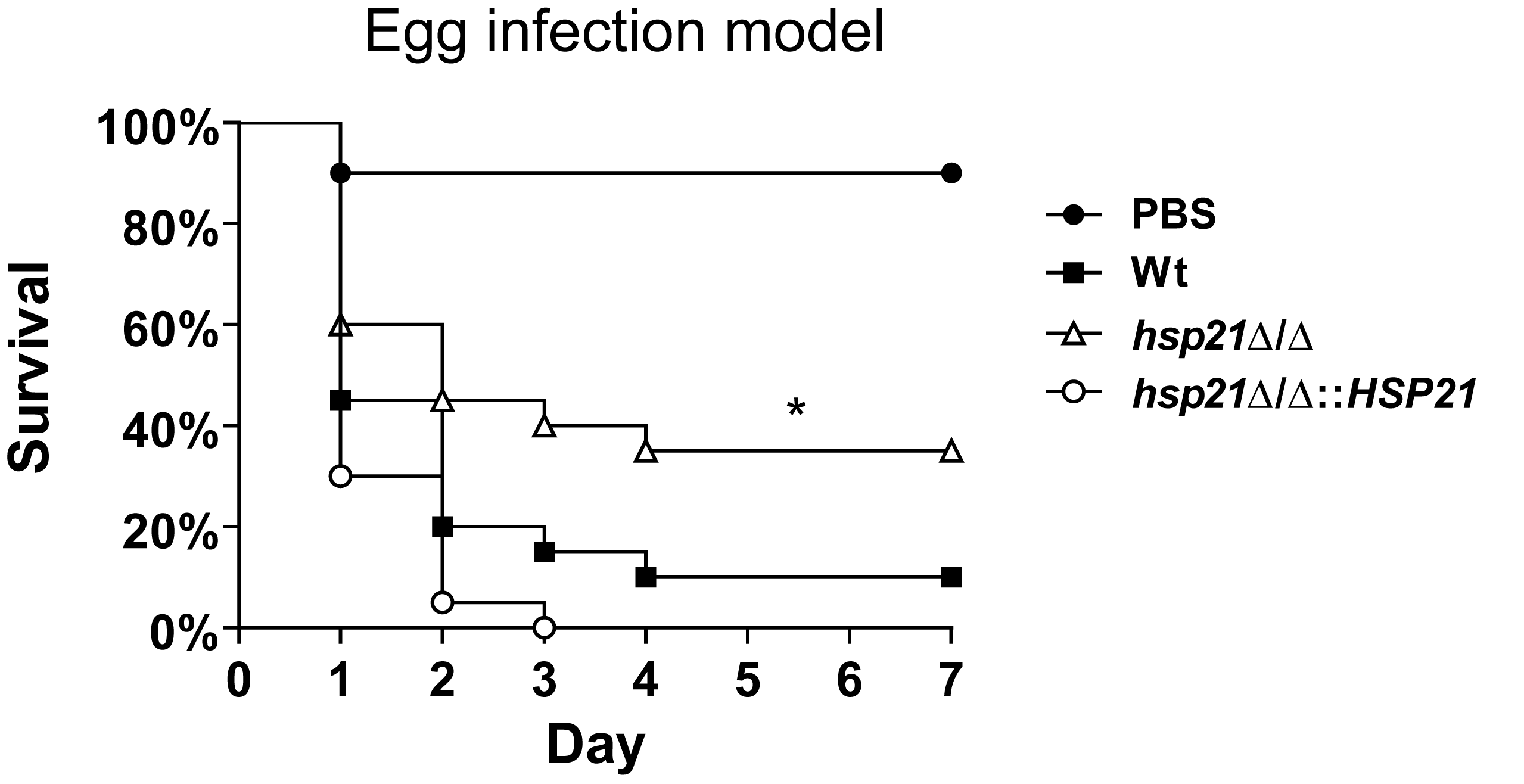

Supplement: Figure S5 — The hsp21 Δ/Δ mutant has attenuated virulence in an embryonated egg infection model. 10-day old embryonated hen eggs were infected with either the wild type (Wt), the hsp21Δ/Δ mutant or the hsp21Δ/Δ::HSP21 complemented strain (n = 20 eggs per C. albicans strain). Survival of the eggs was then monitored daily by candling for a total of 7 days. Results are the mean of at least two independent experiments per strain. *P<0.0001 compared with eggs either infected with the wild type or hsp21Δ/Δ::HSP21 complemented strain. (TIF) [file pone.0038584.s005.tif]
