# Supplementary material for: Small but Crucial: The Novel Small Heat Shock Protein Hsp21 Mediates Stress Adaptation and Virulence in Candida albicans
Source: PLoS One. 2012 Jun 7;7(6):e38584. doi: 10.1371/journal.pone.0038584 (PMC3369842; doi:10.1371/journal.pone.0038584)
Supplement: Table S1 — Transcriptional regulation of C. albicans orf19.822 (HSP21). (DOC) [file pone.0038584.s006.doc]

**Table S1.** *C. albicans* *orf19.822* transcriptional regulation.

| **Study** | **Condition** | **Orf19.822 regulation** | **Reference** |
| --- | --- | --- | --- |
| Heat shock | 23-37°C | 10.9 | [1] |
|  | 30-42°C | 19.0 | [2] |
|  | 30-45°C | 25.0 | [3] |
| Weak acid stress | 300 mM acetic acid (5 h) | 88.7 | [4] |
| Oxidative stress | 0.4 mM H_2_O_2_ | 3.2 | [5] |
| Macrophage | 1 h | 21.9 | [6] |
|  | 2 h | 10.1 | [6] |
|  | 3 h | 29.6 | [6] |
|  | 4 h | 6.8 | [6] |
|  | 6 h | 0.1 | [6] |
| Human blood | Whole blood (30 min) | 4.8 | [7] |
|  | PMN (30 min) | 6.2 | [7] |
|  | MNC (30 min) | 1.4 | [7] |
|  | Plasma (30 min) | 0.3 | [7] |
| Mouse liver | 0 h | 1.0 | [8] |
|  | 30 min | 2.1 | [8] |
|  | 3 h | 5.3 | [8] |
|  | 5 h | 20.0 | [8] |

1. Enjalbert B, Nantel A, Whiteway M (2003) Stress-induced gene expression in *Candida albicans*: absence of a general stress response. Mol Biol Cell 14: 1460-1467.

2. Enjalbert B, MacCallum DM, Odds FC, Brown AJ (2007) Niche-specific activation of the oxidative stress response by the pathogenic fungus Candida albicans. Infect Immun 75: 2143-2151.

3. Nicholls S, Leach MD, Priest CL, Brown AJ (2009) Role of the heat shock transcription factor, Hsf1, in a major fungal pathogen that is obligately associated with warm-blooded animals. Mol Microbiol 74: 844-861.

4. Ramsdale M, Selway L, Stead D, Walker J, Yin Z, et al. (2008) *MNL1* regulates weak acid-induced stress responses of the fungal pathogen *Candida albicans*. Mol Biol Cell 19: 4393-4403.

5. Enjalbert B, Smith DA, Cornell MJ, Alam I, Nicholls S, et al. (2006) Role of the Hog1 stress-activated protein kinase in the global transcriptional response to stress in the fungal pathogen Candida albicans. Mol Biol Cell 17: 1018-1032.

6. Lorenz MC, Bender JA, Fink GR (2004) Transcriptional response of *Candida albicans* upon internalization by macrophages. Eukaryot Cell 3: 1076-1087.

7. Fradin C, De Groot P, MacCallum D, Schaller M, Klis F, et al. (2005) Granulocytes govern the transcriptional response, morphology and proliferation of *Candida albicans* in human blood. Mol Microbiol 56: 397-415.

8. Thewes S, Kretschmar M, Park H, Schaller M, Filler SG, et al. (2007) *In vivo* and *ex vivo* comparative transcriptional profiling of invasive and non-invasive *Candida albicans* isolates identifies genes associated with tissue invasion. Mol Microbiol 63: 1606-1628.
