# Supplementary material for: Small but Crucial: The Novel Small Heat Shock Protein Hsp21 Mediates Stress Adaptation and Virulence in Candida albicans
Source: PLoS One. 2012 Jun 7;7(6):e38584. doi: 10.1371/journal.pone.0038584 (PMC3369842; doi:10.1371/journal.pone.0038584)
Supplement: Table S2 — Primers used in this study. (DOC) [file pone.0038584.s007.doc]

**Table S2.** Primers used in this study.

| **Primer** | **Sequence** | **Reference** |
| --- | --- | --- |
| HSP21-FG | atggttttgtaaaagtttttctcttcatcaatcaacatcttttttgactacattctaataaaacaacaactaatatattattcaattaaatatcgctgctaaaagaagcttcgtacgctgcaggtc | This study |
| HSP21-RG | aaatcaaaaacaaaccaaaaacaaataaagggggttaaaactcaaacaatacttttgaatagaatgaaatctgctcacaaaaaaacatcattctcaaatgccaatctgatatcatcgatgaattcgag | This study |
| HSP21-F1 | gcaatatgatcatgatcatgatcatgc | This study |
| HSP21-R1 | cccccatacaaacaatatttacatatc | This study |
| HSP21-F2 | gtaaatattgtttgtatgggggag | This study |
| HSP21-R2 | gagaaaataaccggttatgctcg | This study |
| HSP21rec-F1 | cactcctgagtgaaagcttgctttgc | This study |
| HSP21rec-R1 | cctcccccatacaagctttatttac | This study |
| ARG4-F1 | ggatatgttggctactgatttagc | [1] |
| ARG4-R1 | aatggatcagtggcaccggtg | [1] |
| HIS1-F1 | ggacgaattgaagaaagctggtgcaaccg | [1] |
| HIS1-R1 | caacgaaatggcctcccctaccacag | [1] |
| URA3-F2 | ggagttggattagatgataaaggtgatgg | [1] |
| RPF-F1 | gagcagtgtacacacacacatcttg | [1] |

Underlined nucleotides of primer HSP21-FG and HSP21-RG indicate pFA-*ARG4* and pFA-*HIS1* annealing regions, and underlined nucleotides of primer HSP21rec-F1 and HSP21rec-R1 indicate *Hind*III restriction sites.

1. Martin R, Hellwig D, Schaub Y, Bauer J, Walther A, et al. (2007) Functional analysis of Candida albicans genes whose Saccharomyces cerevisiae homologues are involved in endocytosis. Yeast 24: 511-522.
